# Climatic control on the location of continental volcanic arcs

**DOI:** 10.1038/s41598-022-26158-2

**Published:** 2022-12-22

**Authors:** Veleda A. P. Muller, Pietro Sternai, Christian Sue, Thibaud Simon-Labric, Pierre G. Valla

**Affiliations:** 1 Dipartimento di Scienze dell’Ambiente e della Terra (DISAT), Università degli Studi di Milano-Bicocca, Piazza della Scienza, 4, Milan, Italy; 2 Institut des Sciences de la Terre (ISTerre), Université Grenoble Alpes, Université Savoie Mont Blanc, CNRS, IRD, IFSTAR, Université Gustave Eiffel, 38000 Grenoble, France; 3 Université de Franche-Comté, 25000 Besançon, France; 4 Centre de Géologie Oisans Alpes, Musée des Minéraux, 38520 Bourg-d’Oisans, France

**Keywords:** Geodynamics, Geology, Geomorphology, Tectonics, Volcanology

## Abstract

Orogens and volcanic arcs at continental plate margins are primary surface expressions of convergent plate tectonics. Although it is established that climate affects the shape, size, and architecture of orogens via orographic erosion gradients, the ascent of magma through the crust and location of volcanoes along magmatic arcs have been considered insensitive to erosion. However, available data reveal westward migration of late-Cenozoic volcanic activity in the Southern Andes and Cascade Range where orography drives an eastward migration of the topographic water divide by increased precipitation and erosion along west-facing slopes. Thermomechanical numerical modeling shows that orographic erosion and the associated leeward topographic migration may entail asymmetric crustal structures that drive the magma ascent toward the region of enhanced erosion. Despite the different tectonic histories of the Southern Andes and the Cascade Range, orographic erosion is a shared causal mechanism that can explain the late-Cenozoic westward migration of the volcanic front along both magmatic arcs.

## Introduction

Volcanic arcs at convergent plate margins are located above the zone of dehydration of a subducting oceanic plate, leading to partial melting of mantle rocks at depths around ~ 100 km, depending on the mean slab dip angle, convergence velocity and thermal structure of the slab and mantle wedge^1–4^. Assuming a vertical magma transfer from the mantle melting zone to the upper crust in subduction settings^4^, slab rollback and trench retreat lead to arc migration towards the subducting plate, whereas slab flattening and trench advancement force the migration of the arc front towards the continent^3,5,6^. In addition to processes that change the depth and position of the mantle melting zone, lithospheric brittle-ductile shear zones serve as fundamental conduits to drive the magma ascent to the surface, thereby affecting the location of the volcanic arc front with respect to the magma source in the mantle and crust^7–10^. The evolution of lithospheric brittle-ductile structures, in turn, depend on the erosion patterns, besides several other factors such as inherited structures, lithology, and convergence rate^11–13^. As a general rule, under the same tectonic conditions, low erosion rates allow forming high and wide orogenic plateaus, whereas high erosion rates imply the formation of smaller orogenic wedges. The climate-tectonics feedbacks also involve orography such that, if the orogen acts as a barrier to atmospheric circulation, enhanced precipitation and erosion on upwind slopes force a leeward migration of the topography^11,13,14^. Although the climatic control on the overall orogenic architecture via surface processes has been largely investigated, its control on rock melting and magma transfer, recognized in extensional settings^15–18^, has been essentially overlooked in convergent continental margins. Here, we propose that an orographic leeward migration of the topographic load may entail the upwind migration of the volcanic arc front due to formation of asymmetric crustal shear zones that allow for magma ascent through the crust, a mechanism that explains observations along the northern and southern American Cordillera.

### The Cascade Range and southern Andes case studies

**Figure 1:**
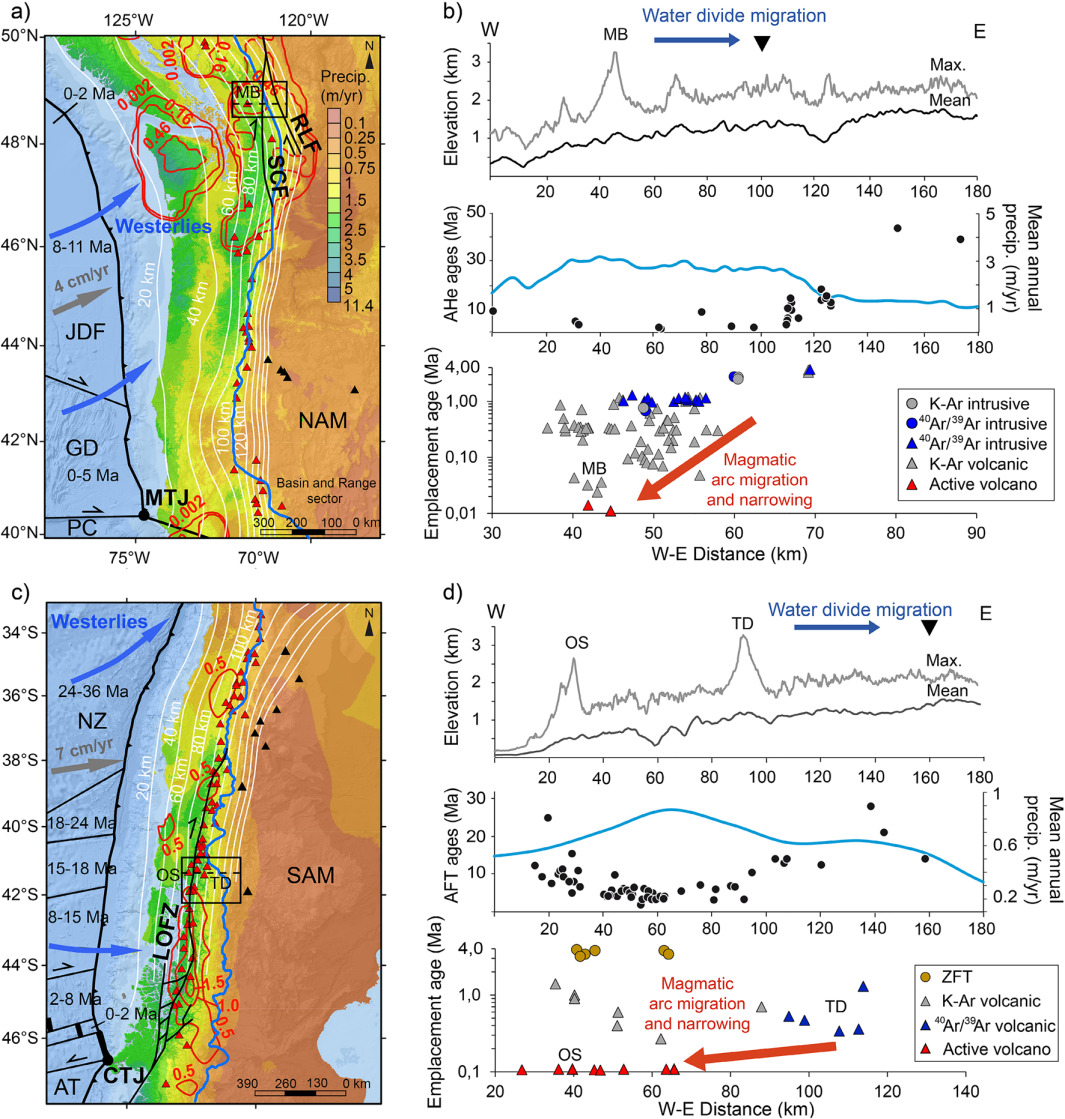
Data compilation from the Cascade Range and the Southern Andes. (**a**, **c**) Maps of the Cascade Range and Southern Andes with mean annual precipitation^36^ and approximated direction of westerlies winds (blue arrows), Quaternary volcanoes^60^ (red triangles: arc volcanoes; black triangles: within-plate volcanoes^21,47^), convergence velocity (gray arrows)^21,41^, isodepths of the top of the slab^43^ (white lines), Quaternary exhumation rates^40,58^ (red lines), topographic water divide (blue line), subduction trench (black line) and the age range of oceanic plates^25,45^. The insets show where the thermochronological data plotted in (b, d) were compiled (Supplementary Table 1a–d) and the dashed lines show the location of the east–west profiles through the Mt. Baker (MB) in CVA, and Osorno (OS) and Tronador (TD) volcanoes in SVZ. Abbreviations: AT: Antarctic Plate; NZ: Nazca Plate; SAM: South American Plate; NAM: North American Plate; JDF: Juan de Fuca Plate; GD: Gorda Plate; PC: Pacific Plate; CTJ: Chile Triple Junction; MTJ: Mendocino Triple Junction; LOFZ: Liquiñe-Ofqui Fault Zone; SCF: Straight Creek Fault, RLF: Ross Lake Fault. (**b**, **d**). Top panels show the mean and maximum elevations (water divide location shown by the black downward triangle, blue arrows show the direction of migration of the topographic water divide). Central panels show the mean annual precipitation rates and apatite (U-Th)/He (AHe) and fission tracks (AFT) ages (Supplementary Table 1a, c and references therein). Bottom panels show emplacement ages of intrusive and volcanic rocks based on K–Ar and ^40^Ar/^39^Ar in volcanic and intrusive rocks, and zircon fission tracks (ZFT) in intrusive rocks (Supplementary Table 1b, d and references therein), red arrows represent the main sense of volcanic arc migration/narrowing.

The Cascade Range and Volcanic Arc (CVA) are generated by the subduction of the oceanic Juan de Fuca Plate (JDF), a remnant of the larger and long-lived Farallon Plate, beneath the western margin of the North American continent between latitudes 40–50°N (Ref.^19,20^). Current subduction occurs at an average rate of ~ 4 cm/yr, increasing from ~ 3 to ~ 4.5 cm/yr from south to north^21^. The subducting plate is ~ 10 Ma old in its central part at ~ 45°N and rejuvenates toward its northern and southern edges (Fig. 1a) providing an example of young, hot and buoyant subducting slab^19–25^. Resistance to subduction and break-up of the JDF Plate into the Explorer and the Gorda microplates is therefore increased since the last ~ 6 Ma in the north and south respectively^21,26,27^. Asthenospheric upwelling is proposed where break-up between the JDF and the Explorer plates occurs accommodated by the transform Nootka Fault entering into subduction^21,27^, and along the southern edge of the JDF plate, nearby the Mendocino Triple Junction^19,21^. Emplacement of volcanic and plutonic rocks in the North Cascades (48–50°N) occurs through compressive and transcurrent shear zones, such as the Straight Creek and Ross Lake fault, that accommodate the accretion of allochthonous terranes since the Mesozoic^28–30^. These shear zones were suggested as preferential conduits for the emplacement of the Chilliwack Batholith, which crystallized from ~ 35 to ~ 4 Ma and is considered of the same magmatic origin as the Quaternary Mt. Baker volcano^23,31–34^ (transect Fig. 1a, b). The volcanic arc in the North Cascades migrates from the northeast towards the southwest since at least ~ 4 Ma. If one considers the intrusive rocks of the Chilliwack Batholith the onset of such trend can be dated back to ~ 12 Ma (Refs.^23,31–33^). Conversely, in the High Cascades (40–48°N) volcanic rocks erupted since the late Miocene are preserved, providing the record of an eastward migration of the arc front^23,31–34^ in a predominantly transtensional tectonic regime that extends into the Basin and Range sector^19,20,34,35^.

The North Cascades interact with westerly Pacific winds, which generate ten times higher precipitation rates on western slopes with respect to the eastern side of the orogen at latitudes where the westerlies are perpendicular to the orogenic belt^36^ (north of ~ 46° N, Fig. 1a). This precipitation pattern is consistent with focused rock exhumation in the western flank of the North Cascades since ~ 10 Ma (Fig. 1b), suggesting that orographic effects are long term features in western North America^11,14,37–40^. While Mesozoic to Tertiary plutonic rocks and metamorphic basement are exhumed in the North Cascades due to orographic erosion, Tertiary to Quaternary volcanics are still preserved in the High Cascades^23,30,31,33,37^. Accordingly, the topographic water divide north of ~ 46°N is 40–70 km more to the east and the average elevation is lower by about 500–1000 m than in the southern sector (Fig. 1a, b). The water divide migration is supposed to have occurred during the Plio-Quaternary^14^. Quaternary volcanoes north of ~ 46°N commonly overlie slab isodepths < 100 km and are systematically located west of the topographic water divide (Fig. 1a). Quaternary volcanoes south of ~ 46°N, instead, overlie slab isodepths ≥ 100 km and are found along the topographic water divide.

The Southern Andes Volcanic Zone (SVZ) is generated by the subduction of the Nazca oceanic plate below the western margin of the South American continent between latitudes 33–46°S (Fig. 1c). Subduction occurs at an average rate of ~ 7 cm/yr (Ref.^41^) with an average slab dip angle of ~ 25° (Refs.^42,43^) since the late Miocene^44^. The age of the subducting oceanic plate decreases southward from ~ 40 Ma to present-day where the Chile Ridge is currently entering into subduction at the Chile Triple Junction (CTJ, Ref.^45^). In the southern Central Andes (33–38°S) several fold-and-thrust belts accommodate deformation and volcanic emplacement since at least the Miocene^44,46,47^. In the northern Patagonian Andes (38–46°S) the general compressive tectonic regime changes to transpressive accommodated by the major strike-slip Liquiñe-Ofqui Fault Zone (LOFZ) active since at least ~ 5 Ma, with denudation ages between ~ 16–10 Ma suggesting an earlier activation^48–51^. In this sector, the Patagonian Batholith is exposed, which allows for reconstructions of the magmatic arc migration since the Cretaceous^52^. Conversely, batholiths are not exposed in the Central Andes where deformed volcanic and sedimentary rocks are preserved at the surface^44,46^. Eruption and emplacement ages of volcanic and intrusive rocks south of 40°S suggest a westward migration and narrowing of the southern volcanic front by around 50 km into the region of the LOFZ since at least the Pleistocene^47,53–55^ (e.g., Tronador-Osorno transect, Fig. 1c, d).

Similarly to the Cascade Range, south of 40°S the SVZ interacts with westerly Pacific winds perpendicular to the orogenic belt associated with significantly increased precipitation on the western slopes of the Southern Andes^36^ (Fig. 1c). Exhumation ages younger than ~ 10 Ma are recorded in the western part of the orogen, whereas systematically older exhumation ages are recorded on the drier eastern peaks of the orogen^49,50,56^ (Fig. 1d). An abrupt increase in exhumation rates since ~ 7 Ma is recorded in the Patagonian Andes, pinpointing the onset of Andean glaciations^40,49,56–58^. An erosion hotspot at 42–46°S is observed in thermochronologic data 2 ± 2 Ma (Fig. 1c, Ref.^58^). In addition, an overall decrease in mean elevation from about 3 km to 1 km and an ~ 70 km eastward migration of the topographic water divide further testify the long-term regional orographic effects on the Andean topography^56,59^. Quaternary stratovolcanoes overlie slab isodepths ≤ 100 km south of 40°S and are systematically west of the topographic water divide (Fig. 1c) whereas, north of 40°S, they overlie slab isodepths > 100 km and are found along the main topographic divide.

The observed topographic leeward and volcanic arc front windward migrations, shared by both the CVA and SVZ despite the different tectonic history, suggest a potential climatic control via erosion on the long-term evolution and location of these continental volcanic arcs. Hereafter, we assess the plausibility of a coupling between crustal magma transfer and climate-controlled erosion.

### Coupled geodynamic and erosion numerical modeling

**Figure 2:**
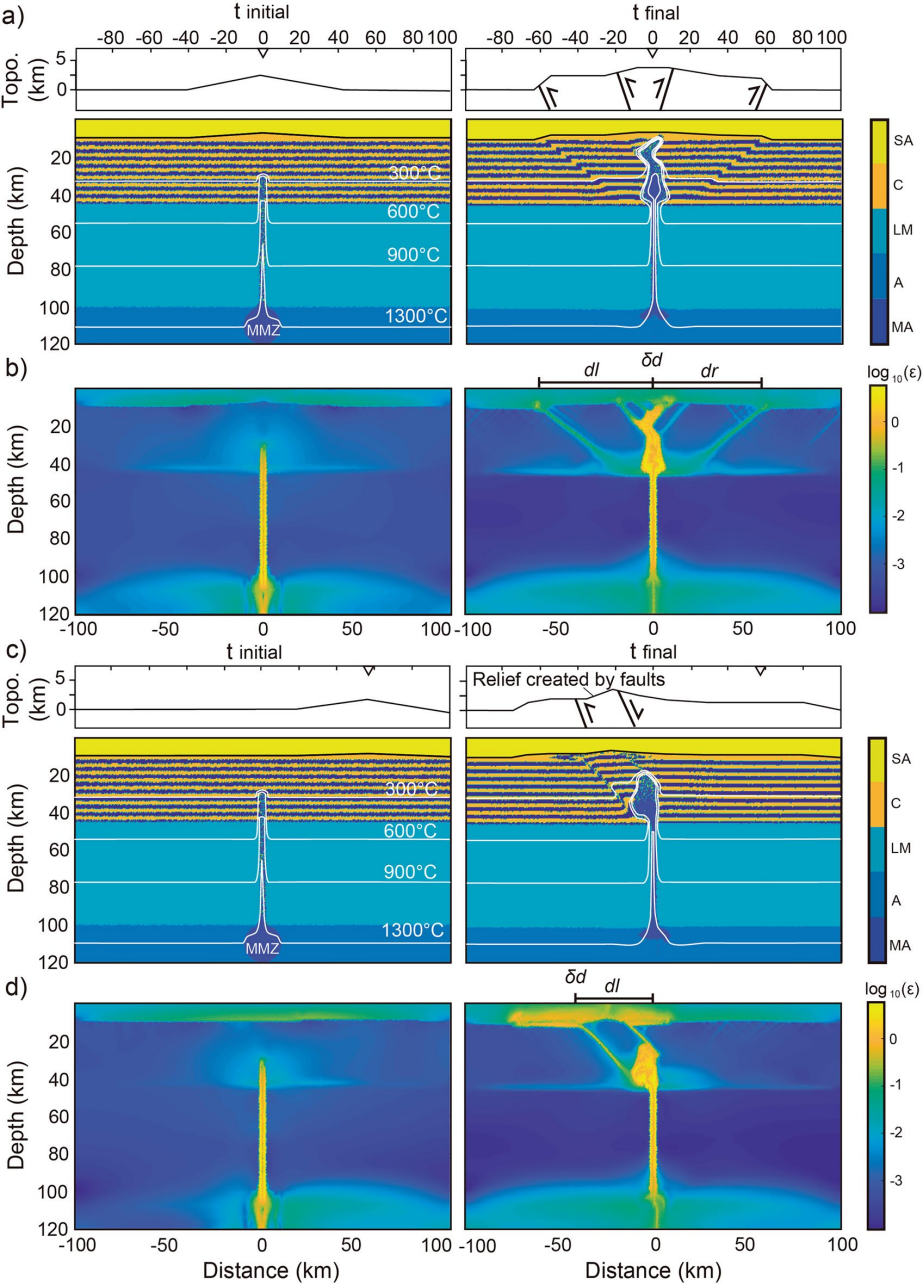
Numerical outcomes of the first set of simulations (Ch = 35 km, Lh = 100 km, Dmc = 20 km). (**a**, **c**) Initial and final lithologic and thermal distribution of the reference simulations with initial central and lateral topography. The color layering in the crust shows the deformation and the vertical exaggeration of the topographic profiles is 3x. (**b**, **d**) Initial and final cumulative bulk strain of the same numerical simulations shown in **(a, c)**. Note the final symmetric system of brittle-ductile structures accommodating the rise of magma to near the surface with initial central topography (**a**, **b**). Note the final asymmetric system of brittle-ductile structures, leading to left-verging magma ascent with initial lateral (shifted rightward) topography (**c**, **d**). $\delta d=dr-dl$, where $dr$ and $dl$ are defined in the text. SA—sticky air; C—crust; LM—lithospheric mantle; A—asthenosphere; MA—molten asthenosphere; MMZ: mantle melting zone.

**Figure 3:**
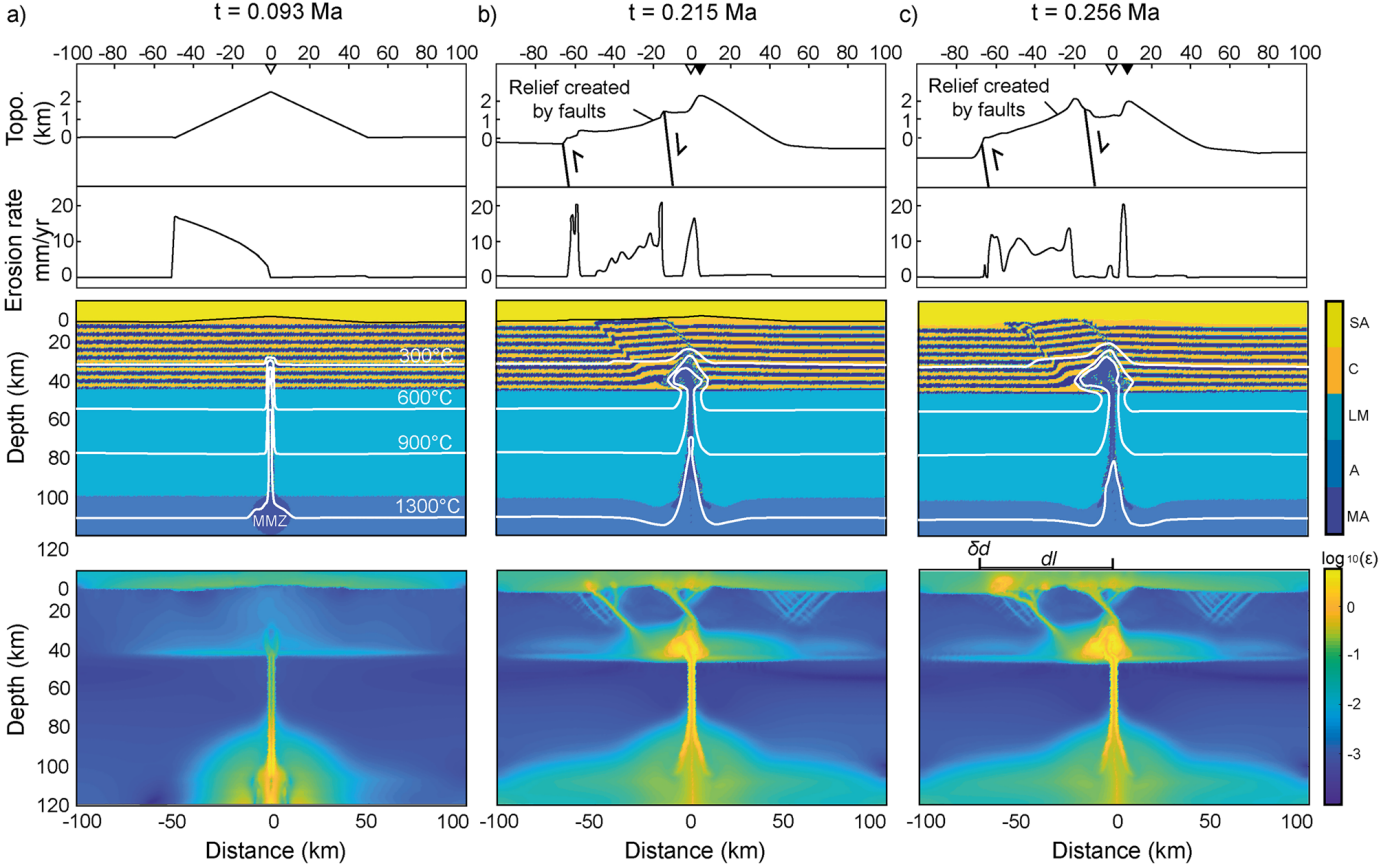
Initial, intermediate and final steps of selected numerical simulation with asymmetric erosion from the second set of simulations (Ch = 35 km, Lh = 100 km, Dmc = 20 km). Topographic profile (8 × vertical exaggeration) and erosion rates (top panels), lithologic and thermal distribution (middle panels), and cumulative bulk strain (lower panels) of the initial state with central topography (**a**), early transient state (**b**), and advanced transient state (**c**). Note the initial water divide (white downward triangle) migrating to the right throughout the simulation (black downward triangle) due to asymmetric erosion and the final asymmetric system of brittle-ductile structures, leading to lateral magma ascent toward the opposite side of the initial topography. Abbreviations are the same as in Fig. 2.

We use a thermo-mechanical (visco-elasto-plastic) geodynamic numerical model coupled to the stream power erosion model (see Methods) to test the hypothesis of an upwind (i.e., westward) migration of the magma ascent and volcanic arc front forced by orography-driven topographic change. The model accounts for the continental crust, upper mantle, asthenosphere (rheological properties are shown in Supplementary Table 2) and a wedge-shaped topography up to 2.5 km high. In a first set of numerical experiments, we impose the initial topography directly above or shifted to the right with respect to a central mantle melting zone (MMZ), analogue to the region where partial melting is generated at ~ 100 km depth feeding crustal magma reservoirs and surface volcanoes^1–4^ (Fig. 2 and Supplementary Figs. 1–4), and no erosion is imposed. This first set of numerical experiments allows us to assess changes in the magma ascent prior to and after the topographic landscape has adjusted to an orographic erosional gradient. In a second set of simulations we impose asymmetric surface erosion, with stream power erosion rates being one order of magnitude higher on left-facing slopes than on right-facing slopes. In these experiments, we consider an initial topography directly above the MMZ (Fig. 3 and Supplementary Figs. 5–8), consistently with observed exhumation rate patterns from the CVA^14,37–40^ and SVZ^40,49,56–58^. The local drainage areas and topographic slopes, continuously adjusting to the progressive crustal deformation due to the emplacement of magma, modulate the erosion rates^11,61^ (see Eq. 13 in Methods), allowing us to address the transient evolution of magma ascent as the topography adjusts to orographic erosion. In order to isolate the effects of climate-driven orography over the crustal trajectories of magma emplacement and ascent towards the surface, we did not impose any tectonic convergence, subduction of oceanic plate, crustal accretion nor sedimentation in these experiments, which simply evolve by upwelling of buoyant magma into the crust until depletion of the MMZ over about 0.3 Ma. This is obviously an oversimplification, but it reduces the degree of complexity of the model and removes any effect from poorly constrained parameters (e.g. spatial–temporal changes in convergence rate or thermal/mechanical/rheological properties). Although this approach does not allow to reproduce the geologic history of the CVA and SVZ in our simulations, it enables us to explore plausible orographic effects on the joint evolution of the topographic landscapes, crustal structures and magma ascent observed in these settings, that is our ultimate goal. We obviously bear this limitation in mind when framing our results in the contexts of the CVA and SVZ.

The model domain extends over 200 × 120 km (horizontal, *x*, and vertical, *y*, dimensions), resolved by 201 × 61 grid points respectively, and is distributed on an irregular Eulerian grid that accounts for a maximum resolution of 1 km along the x and y directions in the upper-central part of the model domain. 400 × 400 Lagrangian markers are randomly distributed along the *x* and *y* dimensions and used for advecting the material properties^16,62,63^ (Supplementary Table 2). The material properties carried by Lagrangian markers are then interpolated onto the Eulerian grid via a 4th order Runge–Kutta interpolation scheme^62,63^. An internal free surface is simulated through a 10 -km thick layer of sticky air^62^. The velocity boundary conditions are free slip at all boundaries (x = 0 and x = 200 km; y = 0 and y = 120 km). The initial temperature gradient is piece-wise linear resulting from an adiabatic temperature gradient of 0.5 °C/km in the asthenosphere^64^ and thermal boundary conditions fixed at 0 °C at the surface and 1327 °C at the lower boundary, with nil horizontal heat flux across the vertical boundaries^63^. Temperature is set to 1327 °C in the MMZ, that is initially circular with 20 km diameter and imposed in the center of the model domain at 100 km depth, consistent with estimates from the CVA^21,65^ and SV^1,66,67^. The upward transfer and emplacement of magma into the crust occurs by buoyancy through a 3 -km wide magmatic channel, which allows for a simplified representation of magmatic percolation by hydrofractures, diffusion, porous flow, and reactive flow through the rheologically stronger mantle lithosphere^63^. The bulk composition of the MMZ is ultramafic, but enrichment by 25% mafic melt is prescribed in the magmatic channel^63^. The viscosity, ${\eta }_{ductile}$ (Eq. 3 in Methods), of partially molten rocks (with ξ > 0.1, Eq. 10 in Methods) is assigned a low constant value of 10^16^ Pa s, and the ${\eta }_{ductile}$ upper limit is set to 10^25^ Pa s (Refs.^16,63,64^). The topographic wedge measures 2.5 km in simulations with central topography, and 1.5 km in simulations with lateral topography, consistent with the observed orography-related change in mean altitude in the CVA and SVZ (Refs.^38,39,56,59^).

The parametric study focuses on three main parameters. First, the crustal thickness (Ch) varies between 45 and 35 km, consistently with literature values^42,65^. Second, the initial depth of the magmatic channel upper tip (Dmc), which assumes values of 15, 20, and 25 km to account for efficient/inefficient magma transfer through the crust. Values are based on estimates of the depth of the regional brittle-ductile transition (~ 20 km, Refs.^42,51,65^), assuming that the rheology of the upper plate crust exerts a primary control on the efficiency of magma transfer. Third, the thickness of the thermal lithosphere (Lh) varies between 90 and 100 km allowing us to account for variations of the elastic lithosphere between ~ 55 and ~ 65 km, as estimated from the regional geophysical data^42,65^.

## Results

**Figure 4:**
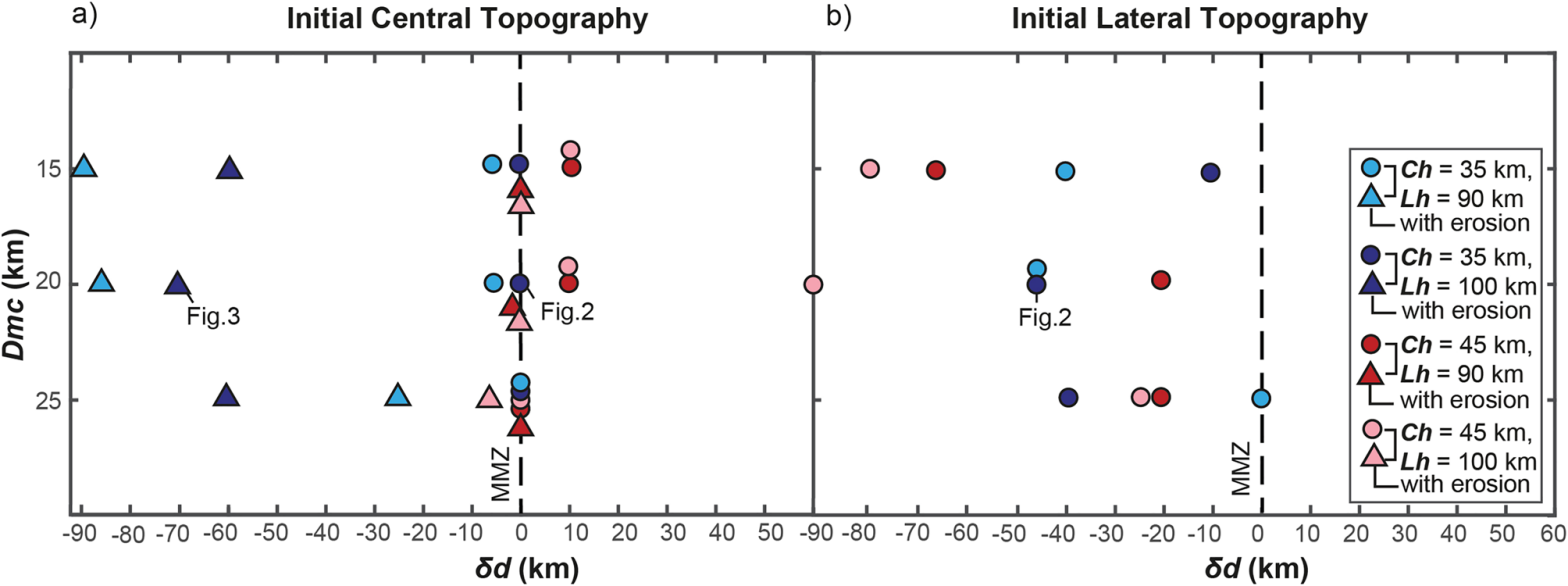
Results of the parametric study. Initial depth of the magmatic channel upper tip (Dmc) vs. $\delta d$ (see text), with initial central (**a**) and lateral (**b**) topography. Triangles in panel (**a**) are the results of simulations with asymmetric erosion (i.e., second set of simulations, see text). Negative values show asymmetry to the left of the mantle melting zone (MMZ) (i.e., central vertical axis of the model domain). Values of Ch and Lh are shown in the legend, cold and hot colors show simulations with thin and thick crust, respectively. See results of simulations in Figs. 2, 3, Supplementary Figs. 1–8, and Table 1.

Results from the first set of numerical experiments show that the emplacement of magma into the crust is accommodated by structures that affect both the brittle upper crust and the ductile lower crust. Simulations that account for an initial central topography allow the formation of symmetric crustal magma reservoirs and structures with respect to the MMZ, feeding volcanoes on both sides of the topographic wedge (Fig. 2a, b and Supplementary Figs. 1–4). Simulations that account for an initial lateral topography develop highly asymmetric crustal magma reservoirs and structures, feeding volcanoes on the opposite side of the initial topographic wedge (Fig. 2c, d and Supplementary Figs. 1–4). We define the degree of asymmetry of the resultant structures (shown by the cumulative bulk strain in Figs. 2b, d and 3) as $\delta d=dr-dl$, where $dr$ and $dl$ are the maximum horizontal distance between the center of the model domain and the surface fault tips to the right and left, respectively. Increasing positive and negative $\delta d$ values indicate increasing asymmetry toward the right and left of the system, respectively (results in Table 1 and Fig. 4). For all tested configurations, a thin crust (Ch = 35 km) and a cold lithosphere (Lh = 100 km) facilitate the brittle strain (Fig. 2 and Supplementary Figs. 1, 2). When the initial topography is lateral, the leftward asymmetry of the strain and magma ascent is enhanced (Figs. 2c–d, 3, and Supplementary Figs. 1, 2). When the initial depth of the magmatic channel upper tip is deep (Dmc = 25 km), the viscous strain in the lower crust accommodates the emplacement of magma, inhibiting the formation of brittle upper crustal structures and the rise of magma to the near surface (Supplementary Figs. 1–4). When Dmc is shallow (15 km), the magma rises easily to the surface through brittle structures near the center of the model, resulting in relatively small values of $\delta d$ (Fig. 4 and Supplementary Figs. 1–4). When Dmc is intermediate (20 km), brittle-ductile structures cross through the crust reaching the surface at further distance from the center of the model (Figs. 2–3, and Supplementary Figs. 1, 3, 4). In the reference model (i.e., Ch = 35 km, Lh = 100 km, and Dmc = 20 km, Fig. 2), an initial lateral topography leads to $\delta d\approx -45$ km (i.e., leftward asymmetry), comparable to the observed westward migration of the magmatic arcs in the natural case studies (Fig. 1). We further remark that, in simulations with initial central topography, a symmetric set of thrusts rooted in the region of magma emplacement form a pop-up structure similar to those observed in the northern sector of the SVZ^46,47^. In the simulations with initial lateral topography, the asymmetric thrust verging towards the west is similar to the “Western Patagonian Thrust” proposed by Ref.^51^.

**Table 1:** Results of the parametric study.

Ch (km)	Lh (km)	Dmc (km)	Initial topography	*Orographic Erosion*	*dl* (km)	*dr* (km)	*δd* = *dr *− *dl (km)*
35	90	15	Central	No	65	60	-5
35	90	20	Central	No	70	65	-5
35	90	25	Central	No	10	10	0
35	90	15	Lateral	No	50	10	-40
35	90	20	Lateral	No	50	5	-45
35	90	25	Cateral	No	64	64	0
35	100	15	Central	No	15	15	0
35	100	20	Central	No	65	65	0
35	100	25	Central	No	65	65	0
35	100	15	Lateral	No	15	5	-10
35	100	20	Lateral	No	45	0	-45
35	100	25	Lateral	No	50	10	-40
45	90	15	Central	No	90	100	10
45	90	20	Central	No	3	13	10
45	90	25	Central	No	0	0	0
45	90	15	Lateral	No	90	25	-65
45	90	20	Lateral	No	25	5	-20
45	90	25	Lateral	No	25	5	-20
45	100	15	Central	No	90	100	10
45	100	20	Central	No	90	100	10
45	100	25	Central	No	0	0	0
45	100	15	Lateral	No	90	10	-80
45	100	20	Lateral	No	90	0	-90
45	100	25	Lateral	No	30	5	-25
35	90	15	Central	Yes	93	3	-90
35	90	20	Central	Yes	95	10	-85
35	90	25	Central	Yes	25	0	-25
35	100	15	Central	Yes	70	10	-60
35	100	20	Central	Yes	70	0	-70
35	100	25	Central	Yes	70	10	-60
45	90	15	Central	Yes	10	10	0
45	90	20	Central	Yes	8	5	-3
45	90	25	Central	Yes	0	0	0
45	100	15	Central	Yes	13	5	-8
45	100	20	Central	Yes	5	5	0
45	100	25	Central	Yes	0	0	0

*Ch* Crustal thickness, *Lh* Lithospheric thickness, *Dmc* Initial depth of the magmatic channel upper tip ***dl*** and ***dr*** Maximum horizontal distances between the center of the model domain and the surface fault tips in surface to the left and to the right, respectively; ${\varvec{\delta}}{\varvec{d}}={\varvec{d}}{\varvec{r}}-{\varvec{d}}{\varvec{l}}$ is the degree of the asymmetry of the accumulated bulk strain (negative values show asymmetry to the left).

Results from the second set of simulations show that the progressive rightward migration of the topographic divide leads to negative $\delta d$ values when Ch = 35 km (Figs. 3, 4, and Supplementary Figs. 5, 6). The topography migrates toward the right ~ 20 km in ~ 0.3 Ma (Fig. 3c and Supplementary Figs. 5, 6), roughly consistent with the eastward migration of the water divide by ~ 50–70 km observed in the study regions during the Plio-Quaternary. Uplift of additional topographic peaks on the left side of the model domain occurs due to left-verging thrusts accommodating the magma ascent. We associate this topography to that of volcanic edifices west of the main topographic divide (Fig. 1). When the crust is thick (Ch = 45 km) the viscous strain accommodates almost entirely the magma emplacement and limited bulk strain accumulates along shallow brittle faults that tend to be symmetric (Fig. 3 and Supplementary Figs. 7, 8). Accordingly, no topographic relief is generated on the left side of the model domain and the topography migrates to the right by ~ 10 km in ~ 0.3 Ma (Supplementary Figs. 7, 8).

## Discussion

The break-up of the Farallon plate into the Juan de Fuca and Nazca plates in the early Miocene established a kinematic configuration similar to the present day in terms of slab partitioning and convergence rates along the American Cordillera^20,44^. Although the onset of the eastward migration of the topographic water divide in the CVA and the SVZ is uncertain, systematically younger exhumation ages on the western slopes of both the North Cascades and northern Patagonian Andes (Fig. 1) suggests that orographic effects were already established in the latest Miocene^37–39,56,57^, leading to the formation of the current landform throughout the Pleistocene^14,39,58,59^. Although low-temperature thermochronometric ages constrain the onset of enhanced erosion between ~ 10 and ~ 6 Ma in the North Cascades and the Southern Andes, respectively^37,38,56^, large uncertainties exist about the paleo position of topographic water divide and paleo-elevations in general. A similar issue pertains to the onset of arc front migration, which can be only roughly constrained from batholith ages^31,52^, but precise information about the exact position of the paleo volcanic arc front is lacking. Our modeling results show a fast response of the crustal strain and magmatic plumbing system to orographic erosion, which adjust to topographic changes in just a few hundreds of thousands of years (Figs. 2 and 3).

**Figure 5:**
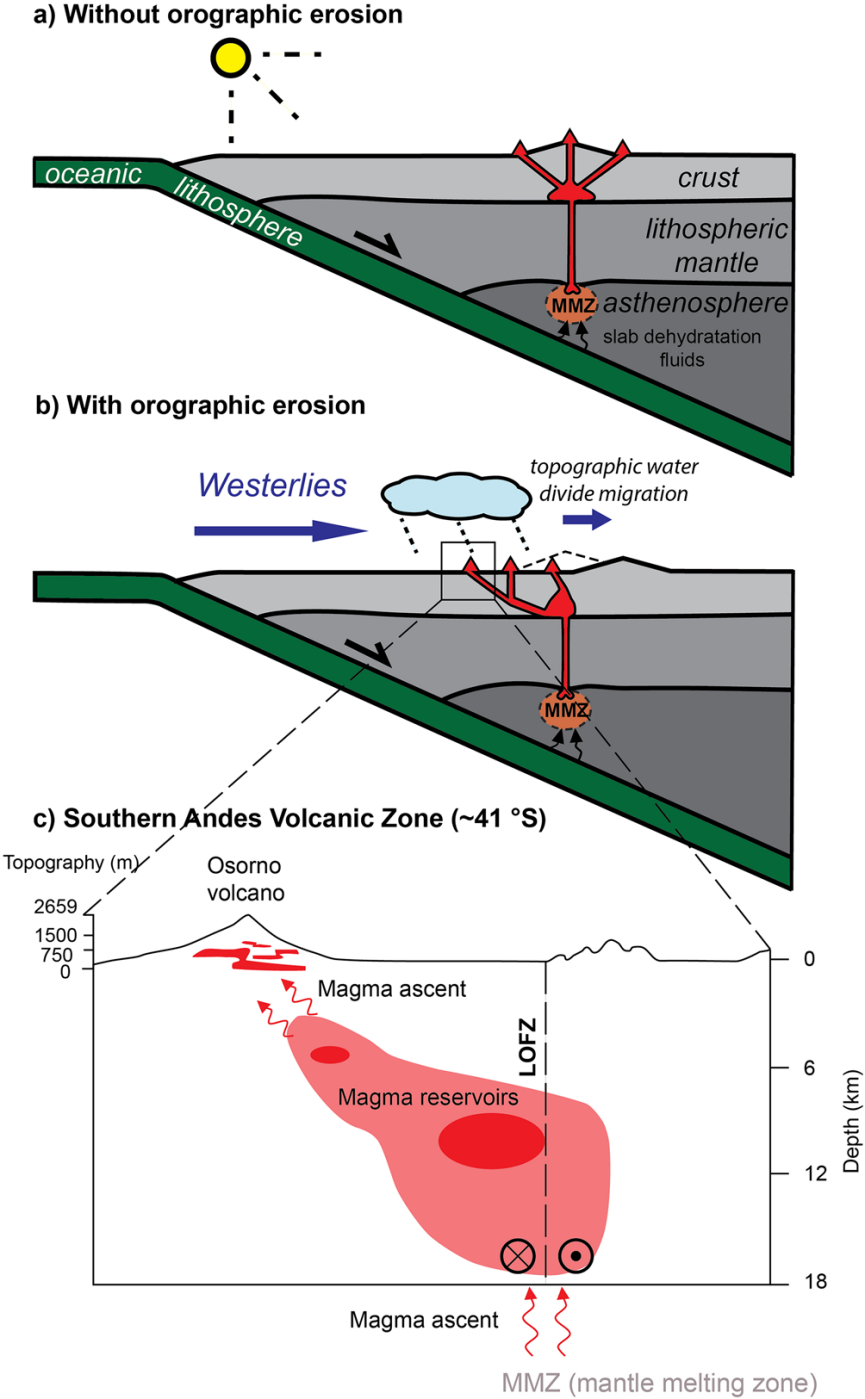
Schematic representation of the orographic forcing on the location of continental volcanic arcs. Cross-sections illustrate the magma ascent from a sub-lithospheric mantle melting zone (MMZ) to surface volcanoes (red triangles) trough newly-formed or reactivated structures. (**a**) Analogy for orogens and volcanic arcs without orographic erosion, and **b**) with orographic erosion gradient. In the latter case, the deformation is enhanced on the windward side and so is the magma ascent through the brittle-ductile structures. (**c**) Schematic representation of the crustal magma reservoirs interpreted from magnetotelluric data underneath the Osorno volcano showing the marked westward magma ascent in agreement with our modeling results and the proposed orographic effect on the location of the volcanic arc front (modified after Ref.^70^).

In the CVA, the present-day clockwise rotation of the Oregon and Washington blocks and the counterclockwise displacement of the volcanic front since the Miocene have been ascribed to differential along strike rollback of the Juan de Fuca slab^20,31,34,35^. However, since the magmatic source follows the rollbacking slab^1,4–6^, this model requires some unjustified degree of decoupling between rollback and arc front migration^34^. In the North Cascades, the increase in the rate of arc front migration towards the southwest since ~ 4 Ma (Refs.^31–33^) requires faster slab rollback rates than during the late Miocene, difficult to reconcile with the transpressive regime and present northeastward displacements of the Washington block^31,34^. The presence of slab-derived magmas (i.e., adakites^68^) in southernmost and northernmost volcanoes of the CVZ may suggest shallower (i.e., closer to the trench) magma production near the breakup of the Juan de Fuca Plate into the Gorda and Explorer plates^22,24,68^. However, if the thermal gradient along the strike of the subduction system set alone the surface location of the volcanic arc, trench-ward migration of the volcanic front would be observed both in the northern and southern sectors of the CVA. Thus, the opposite sense of arc front migration in the North Cascades and in the High Cascades^34^ can hardly be explained by along-strike slab age and/or thermal changes alone. Since Quaternary volcanoes are emplaced over fault systems, orographic interactions with westerly Pacific winds (Fig. 1a, b) and the mechanistic link between asymmetric erosion and crustal structures accommodating the magma ascent (Figs. 2, 3, 4 and 5) appear as a suitable along-strike differential forcing to drive the observed latitudinal arc migration front trend reversal. Magnetotelluric data (Fig. 5) show westward dipping magmatic plumbing systems in the North Cascades^69^, consistent with our modeling results (Figs. 2c, d and 3) and an orographic control on the location of volcanic arc front.

In the Southern Andes, a Plio-Quaternary magmatic arc narrowing toward the west has been related to the steepening of a formerly shallow slab^55^ or a decrease in convergence velocity^54^. South of ~ 40°S, Plio-Pleistocene strain localization along the transpressive system of the LOFZ has been ascribed to oblique convergence of the Nazca Plate and the CTJ approaching its current position^48–50^, driving the ascent of magmas and setting the observed trench-ward deflection of the SVZ^8,48,49,54^ (Fig. 1c). Geophysical imaging of the deep crust and mantle show that the crustal magma reservoirs of the Osorno volcano are located up to 10 km east of the volcanic edifice^70^, consistent with our modeling results and hypothesis of focused orographic erosion facilitating the strain and magma ascent across the windward side of orogens^11–13^ (Fig. 5). Field evidence regarding the presence of the west vergent “Western Patagonian Thrust”^51^ reinforces our proposal of asymmetric faults assisting magma ascent towards the west. Also in agreement with previous studies^8^, our models indicate that orographic erosion of a thin crust facilitates the formation of major structures, suggesting that orographic erosion may have contributed to the activation, or re-activation, of the LOFZ. Heat advection from the younger slab and the CTJ in the south can likely enhance this effect. The Nazca Plate is younger toward its southern edge (CTJ) and thus the thermal regime of the subduction is likely higher in the northern Patagonian Andes^53,67^. However, no slab-derived magmas are found in the whole SVZ^8,52,66,71^ and the depth of the source of partial melting is unlikely to change significantly along the strike of the belt^1,52,67^. Differences in magma composition along the strike of the SVZ show a tendency toward more mafic magmas in the south. This trend can be simply explained by a reduced crust-magma interaction due to the thinner crust in this sector^8,66,71^, which also promotes the orographic forcing on the magma ascent proposed here (Fig. 4).

We propose a common explanation to the westward migration of the volcanic arc fronts in the North Cascades and the northern Patagonian Andes, located several thousands of kilometers apart and in different tectonic/structural settings, by the shared orographic interactions between topography and westerly Pacific winds (Fig. 5). The climatic control on magmatism would occur not only through a forcing from ice building-melting and erosion on the magma production^15,72^, but also through orography and topographic change facilitating crustal strain and magma transfer verging toward the region of enhanced precipitation and erosion. Because volcanic arcs provide a substantial contribution to the evolution of climate across timescales, this recognition provides additional evidence of the tight coupling between climate, surface processes, magmatism, and plate tectonics^73,74^.

## Methods

### Thermomechanical model

The numerical model is based on the finite differences with marker-in-cell technique and incorporates temperature-dependent rheologies for solid and partially-molten rocks^63^. The model accounts for (1) mechanical, (2) visco-elasto-plastic rheological, (3) thermal, and (4) partial rock melting components. A short description of the model components is provided hereafter. Additional details and full description of the numerical techniques are provided elsewhere^16,62,63^.

### Mechanical component

The continuity equation,

1 $$\frac{\partial \rho }{{\partial t}} + \nabla \left( {\rho v } \right) = 0,$$

where $\rho$ is the local density, $t\mathrm{ \: is \: time}, {v}$ is the velocity vector, and $\nabla$ is the divergence operator, allows for the conservation of mass during the displacement of a geological continuum.

The momentum equation,

2 $$\frac{{\partial \sigma_{ij} }}{{\partial x_{i} }} + \rho g_{i} = \rho \left( {\frac{{\partial v_{i} }}{\partial t} + v_{j} \frac{{\partial v_{i} }}{{\partial x_{j} }}} \right),$$

where ${\sigma }_{ij}$ is the stress tensor, ${x}_{i}$ and ${x}_{j}$ are spatial coordinates, and ${g}_{i}$ is the *i*-th component of the gravity vector, describes the changes in velocity of an object in the gravity field due to internal and external forces.

### Rheological component

Ductile deformation is thermally activated and occurs by viscous flow. Diffusion and dislocation creep are computed based on material shear viscosity^62,63^, ${\eta }_{ductile}$, defined as:

3 $$\frac{1}{{\eta_{ductile} }} = \frac{1}{{\eta_{diff} }} + \frac{1}{{\eta_{disl} }},$$

with

$$\eta_{diff} = \frac{{\eta_{0} }}{{2\sigma_{cr}^{n - 1} }}exp\left( {\frac{{E_{a} + PV_{a} }}{RT}} \right),$$

and

$$\eta_{disl} = \frac{{\eta_{0}^{\frac{1}{n}} }}{2}exp\left( {\frac{{E_{a} + PV_{a} }}{nRT}} \right)\dot{\varepsilon }_{II}^{{\frac{1}{n} - 1}} ,$$

where ${\eta }_{diff}$ and ${\eta }_{disl}$ are the shear viscosity for diffusion and dislocation creep, respectively, ${\eta }_{0}$ is the material static viscosity, ${\sigma }_{cr}$ is the diffusion-dislocation transition critical stress, *n* is the stress exponent, ${E}_{a}$ is the activation energy, ${V}_{a}$ is the activation volume, *P* is pressure, *R* is the gas constant, *T* is temperature, and $\dot{{\varepsilon }_{II}}$ is the second invariant of the strain rate tensor. Then, the viscous deviatoric strain rate tensor, $\dot{\varepsilon }^{\prime }_{{ij \left( {viscous} \right)}}$, is computed as:

4 $$\dot{\varepsilon }^{\prime }_{{ij \left( {viscous} \right)}} = \frac{1}{{2\eta_{ductile} }}\sigma^{\prime }_{ij } + \delta_{ij} \eta_{bulk} \dot{\varepsilon }_{kk} = \frac{1}{{2\eta_{diff} }}\sigma^{\prime }_{ij } + \frac{1}{{2\eta_{disl} }}\sigma^{\prime }_{ij } + \delta_{ij} \eta_{bulk} \dot{\varepsilon }_{kk} ,$$

where $\sigma^{\prime }_{ij }$ is the deviatoric stress tensor,${\delta }_{ij}$ is the Kronecker delta, ${\dot{\varepsilon }}_{kk}$ is the volumetric strain rate (e.g., related to phase transformations), ${\eta }_{bulk}$ is the bulk viscosity.

Elastic deformation is reversible and assumes proportionality of stress and strain. The elastic deviatoric strain rate tensor, $\dot{\varepsilon }^{\prime }_{{ij \left( {elastic} \right)}}$, is computed as:

5 $$\dot{\varepsilon }^{\prime }_{{ij \left( {elastic} \right) }} = \frac{1}{2\mu } \frac{{\overset{\lower0.5em\hbox{$\smash{\scriptscriptstyle\smile}$}}{D} \sigma^{\prime }_{ij} }}{Dt},$$

where $$\mu$$ is the shear modulus and $$\frac{{\overset{\lower0.5em\hbox{$\smash{\scriptscriptstyle\smile}$}}{D} \sigma^{\prime }_{ij} }}{Dt}$$ is the objective co-rotational time derivative of the deviatoric stress tensor.

Plastic (or brittle) localised deformation occurs at low temperature in the upper part of the lithosphere, after reaching the absolute shear stress limit, ${\sigma }_{yield}$, is defined as:

6 $$\sigma_{yield} = C + \sin \left( \varphi \right)P,$$

where $C$ is cohesion and $\varphi$ is the effective internal friction angle. The plastic strain rate tensor, $\dot{\varepsilon }^{\prime }_{{ij \left( {plastic} \right)}}$, is then computed as:

7 $$\dot{\varepsilon }^{\prime }_{{ij \left( {plastic} \right) }} = 0 \; \text {for} \;\sigma_{II} < \sigma_{yield} ,\;\dot{\varepsilon }^{\prime }_{{ij \left( {plastic} \right) }} = {\mathcal{X}}\frac{{\partial \sigma^{\prime }_{ij} }}{{2\sigma_{II} }} \; \text {for} \; \sigma_{II} \ge \sigma_{yield} ,$$

where $\mathcal{X}$ is the plastic multiplier which satisfies the plastic yielding condition ${\sigma }_{II} = {\sigma }_{yield}$.

At the lithospheric scale, all deformation mechanisms occur jointly and the overall visco-elasto-plastic rock strain rate tensor, $\dot{\varepsilon }^{\prime }_{{ij\left( {bulk} \right)}}$, is defined as:

8 $$\dot{\varepsilon }^{\prime }_{{ij\left( {bulk} \right)}} = \dot{\varepsilon }^{\prime }_{{ij\left( {viscous} \right)}} + \dot{\varepsilon }^{\prime }_{{ij\left( {elastic} \right)}} + \dot{\varepsilon }^{\prime }_{{ij\left( {plastic} \right)}}$$

### Thermal component

Heat conservation during advective and conductive heat transfer in the continuum is computed by the energy equation:

9 $$\rho C_{P} \frac{DT}{{Dt}} - div\left( {c\nabla T} \right) + v\nabla T = H_{r} + H_{s} + H_{a} + H_{l} ,$$

where $C_{P}$ is specific heat capacity at a constant *P*, $c$ is the thermal conductivity, $H_{r} + H_{s} + H_{a} + H_{l}$ are the volumetric heat productions by radiogenic, shear, adiabatic and latent heat, respectively. $H_{a} \propto \frac{DP}{{Dt}}$, $H_{s} = \sigma^{\prime }_{ij} \dot{\varepsilon }^{\prime }_{{ij\left( {viscous} \right)}}$, and $H_{r}$ and $H_{l}$ are the radiogenic and latent heat productions (defined in Supplementary Table 2).

### Partial melting component

Partial melting occurs between the wet solidus, ${T}_{s}$, and dry liquidus, ${T}_{l}$, of the considered lithologies^75–77^ (Supplementary Table 2). $\xi$ is the volumetric fraction of melt that increases linearly with T at a constant *P* (Ref.^64^), so that,

10 $$\left\{ \begin{gathered} \xi = 0\quad \quad \quad \quad at\;T \le T_{s} \hfill \\ \xi = \frac{{\left( {T - T_{s} } \right)}}{{\left( {T_{l} - T_{s} } \right)}}\;\;at\;T_{s} < T < T_{l} \hfill \\ \xi = 1\quad \quad \quad \quad at\;T \ge T_{l} \hfill \\ \end{gathered} \right.$$

The effective density, ${\rho }_{eff}$, of partially molten rocks is then computed by^16,63^:

11 $$\rho_{eff} { } = { }\rho_{s} { }\left( {{ }1{ }{-}{ }\xi { } + { }\xi \frac{{\rho_{l}^{0} }}{{\rho_{s}^{0} }}{ }} \right),$$

where ${\rho }_{l}^{0}$ and ${\rho }_{s}^{0}$ are the standard densities of solid and molten rocks, respectively. The solid rock density, ${\rho }_{s}$, is calculated as^63^:

12 $$\rho_{s} = \rho_{o} \left[ {1 + \beta \left( {P - P_{0} } \right)\left] { \times } \right[1 {-} \alpha \left( {T - T_{0} } \right)} \right],$$

where $\beta$ is compressibility, $\alpha$ is thermal expansion and ${\rho }_{o}$, ${P}_{0}$, and ${T}_{0}$ are density, pressure and temperature of rocks at surface conditions.

### Erosion model

During the fluvial incision of an uplifting topography the erosion rate, $\dot{e}$, depends primarily on channel slope, river discharge and rock type^61^. An empirical relationship was derived^11,61^, such that:

13 $$\dot{e} = \frac{dz}{{dt}} = kA^{m} \left| {\frac{dz}{{dx}}} \right|^{n}$$

where $z$ is the elevation, $A$ is the basin drainage area taken as a proxy for river discharge, $\left|\frac{dz}{dx}\right|$ is the magnitude of the local topographic slope, and $k$, $m$, and $n$ are empirical parameters, usually determined by fitting models to river longitudinal profiles. While $k$ is used as a scaling constant, different values of m and n lead to differences in the shape of river profiles, but commonly used values (e.g., 0.3 < $m$ < 0.5 and $n$ ≈ 1) predict realistic concave-up profiles such as those observed along bedrock rivers incising uplifting topographies^11,61,78^. Although simplistic, Eq. (13) captures the main physical processes and parameter dependencies and, most important for this study, it provides the feedback mechanisms between tectonic uplift and erosion. Tectonic uplift increases slope, and the rise of topography increases drainage area, both leading to an increase in erosion rates.

Integration on a discrete topography of the top of the lithosphere allows for Eq. (13) to be solved numerically, thereby assessing surface elevation changes in response to the tectonic strain and fluvial incision^11,16^. At each time step, the surface load changes associated with modifications of the modelled landscape are computed. The orographic enhancement of precipitation, and hence fluvial erosion, is included in the surface processes model by assigning different $k$ values (Eq. 13) on negatively (left-facing) and positively (right-facing) sloping topography.

## Supplementary Information

Supplementary Information.

## Data Availability

Supplementary Tables and Figures are available for this paper.
