# Supplementary material for: Climatic control on the location of continental volcanic arcs
Source: Sci Rep. 2022 Dec 22;12:22167. doi: 10.1038/s41598-022-26158-2 (PMC9780350; doi:10.1038/s41598-022-26158-2)
Supplement: Supplementary file 1 — Supplementary Information. [file 41598_2022_26158_MOESM1_ESM.docx]

**Supplementary Data**

**Supplementary Table 1a – Summary of apatite (U-Th)/He ages (AHe) around the Mont Baker, Cascade Range (data inside the inset of Fig. 1a, plotted in the central panel of Fig. 1b).** W-E distance corresponds to the horizontal distance between each sample and the western boundary of the inset in Fig. 1a. In the field “Type”, I = intrusive, V = volcanic, M = metamorphic. Geographic Coordinate System WGS 1984.

| **Method** | **Name** | **Longitude** | **Latitude** | **Elevation** | **W-E distance (km)** | **Age (Ma)** | **Error (Ma)** | **Type** | **Place of sampling** | **Reference** |
| --- | --- | --- | --- | --- | --- | --- | --- | --- | --- | --- |
| AHe | 01-1 | -121.1975 | 48.7287 | 1953 | 112 | 12.4 | 2.7 | I | Skagit Gorge Profile | Simon-Labric et al., 2014 |
| AHe | 01-2 | -121.1981 | 48.7234 | 1758 | 112 | 14.5 | 2.1 | I | Skagit Gorge Profile | Simon-Labric et al., 2014 |
| AHe | 01-4 | -121.2005 | 48.7123 | 1347 | 111 | 9.1 | 1.3 | I | Skagit Gorge Profile | Simon-Labric et al., 2014 |
| AHe | 01-5 | -121.2041 | 48.7112 | 1155 | 111 | 10.1 | 1.4 | I | Skagit Gorge Profile | Simon-Labric et al., 2014 |
| AHe | 01-6 | -121.2072 | 48.7076 | 904 | 111 | 6.4 | 0.6 | I | Skagit Gorge Profile | Simon-Labric et al., 2014 |
| AHe | 01-7 | -121.2083 | 48.70667 | 680 | 111 | 5.9 | 0.6 | I | Skagit Gorge Profile | Simon-Labric et al., 2014 |
| AHe | 02-1 | -121.2092 | 48.6999 | 345 | 110 | 4.3 | 1.0 | I | Skagit Gorge Profile | Simon-Labric et al., 2014 |
| AHe | 02-2 | -121.2116 | 48.6973 | 241 | 110 | 3.5 | 0.8 | I | Skagit Gorge Profile | Simon-Labric et al., 2014 |
| AHe | 03-1 | -121.0988 | 48.8223 | 1967 | 123 | 18.1 | 1.8 | I | Ross Lake Profile | Simon-Labric et al., 2014 |
| AHe | 03-2 | -121.0914 | 48.8182 | 1778 | 124 | 14.3 | 1.4 | I | Ross Lake Profile | Simon-Labric et al., 2014 |
| AHe | 03-3 | -121.0828 | 48.8095 | 1617 | 124 | 15.1 | 1.5 | I | Ross Lake Profile | Simon-Labric et al., 2014 |
| AHe | 03-5 | -121.0703 | 48.7943 | 1181 | 126 | 15.3 | 4.7 | I | Ross Lake Profile | Simon-Labric et al., 2014 |
| AHe | 04-1 | -121.0595 | 48.7793 | 692 | 127 | 11.0 | 2.2 | I | Ross Lake Profile | Simon-Labric et al., 2014 |
| AHe | 04-2 | -121.0612 | 48.7818 | 916 | 127 | 11.3 | 2.0 | I | Ross Lake Profile | Simon-Labric et al., 2014 |
| AHe | 013D25 | -120.6298 | 48.5743 | 1173 | 175 | 38.8 | 7.8 | I | North Washington Cascades | Simon-Labric et al., 2014 |
| AHe | 013D27 | -120.8371 | 48.6243 | 1033 | 152 | 43.7 | 6.9 | I | North Washington Cascades | Simon-Labric et al., 2014 |
| AHe | 013D28 | -121.0771 | 48.7167 | 628 | 125 | 15.6 | 3.3 | I | North Washington Cascades | Simon-Labric et al., 2014 |
| AHe | 09CAS16 | -121.9142 | 48.8797 | 350 | 32 | 3.4 | 0.7 | I | North Washington Cascades | Simon-Labric et al., 2014 |
| AHe | 09CAS18 | -121.9264 | 48.8639 | 854 | 31 | 4.8 | 1.8 | I | North Washington Cascades | Simon-Labric et al., 2014 |
| AHe | 12Cas14 | -121.32201 | 48.63378 | 142 | 98 | 2.4 | 0.6 | I | North Washington Cascades | Simon-Labric et al., 2014 |
| AHe | 12Cas25 | -121.39841 | 48.58596 | 116 | 89 | 2.7 | 0.2 | I | North Washington Cascades | Simon-Labric et al., 2014 |
| AHe | 12Cas26 | -122.20283 | 48.81322 | 73 | 0 | 8.7 | 2.7 | I | North Washington Cascades | Simon-Labric et al., 2014 |
| AHe | 12Cas10 | -121.17249 | 48.70697 | 280 | 115 | 5.9 | 0.8 | I | North Washington Cascades | Simon-Labric et al., 2014 |
| AHe | WC95-32 | -121.5 | 48.67 | 300 | 78 | 8.6 | 0.5 | I | Yellow Aster | Reiners et al., 2002 |
| AHe | LA01A | -121.641 | 48.835 | 1651 | 62 | 1.4 | 0.1 | I | Lake Ann pluton | Reiners et al., 2002 |
| AHe | LA01B | -121.641 | 48.835 | 1651 | 62 | 1.4 | 0.1 | I | Lake Ann pluton | Reiners et al., 2002 |
| AHe | LA01C | -121.641 | 48.835 | 1651 | 62 | 1.2 | 0.1 | I | Lake Ann pluton | Reiners et al., 2002 |
| AHe | LA02A | -121.645 | 48.831 | 1471 | 62 | 2.2 | 0.1 | I | Lake Ann pluton | Reiners et al., 2002 |
| AHe | LA02B | -121.645 | 48.831 | 1471 | 62 | 2.0 | 0.1 | I | Lake Ann pluton | Reiners et al., 2002 |

**Supplementary Table 1b – Summary of isotopic ages <4 Ma of rocks around the Mount Baker, Cascade Range (data inside the inset of Fig. 1a, plotted in the bottom panel of Fig. 1b).** In the field “Method”, **Wr K-Ar:** ^40^K/^40^Ar in whole rock, **Pl Ar-Ar:** ^40^Ar/^39^Ar in plagioclase, **Bt K-Ar:** ^40^K/^40^Ar in biotite, **Hb K-Ar:** ^40^K/^40^Ar in hornblende; **Active volcano:** 0.01 Ma is an arbitrary recent age and the position is according to Venzke (2013). Other columns like in Table 1a.

| **Method** | **Name** | **Longitude** | **Latitude** | **W-E distance (km)** | **Age (Ma)** | **Error (Ma)** | **Rock** | **Type** | **Place of sampling** | **Reference** |
| --- | --- | --- | --- | --- | --- | --- | --- | --- | --- | --- |
| Wr K-Ar | MB-493 | -121.736092 | 48.85944238 | 51.89168666 | 1.191 | 0.094 | Dacite | V | Kulshan caldera suite | Hildreth et al., 2003 |
| Wr K-Ar | MB-500 | -121.840496 | 48.82545378 | 40.28829116 | 1.156 | 0.130 | Andesite | V | Kulshan caldera suite | Hildreth et al., 2003 |
| Wr K-Ar | MB-316 | -121.694892 | 48.81849939 | 56.47058367 | 1.155 | 0.038 | Rhyodacite | V | Kulshan caldera suite | Hildreth et al., 2003 |
| Wr K-Ar | MB-145 | -121.83771 | 48.82812465 | 40.59801732 | 1.060 | 0.010 | Rhyodacite | V | Kulshan caldera suite | Hildreth et al., 2003 |
| Wr K-Ar | MB-212 | -121.70544 | 48.83211299 | 55.29837474 | 1.032 | 0.011 | Rhyodacite | V | Kulshan caldera suite | Hildreth et al., 2003 |
| Wr K-Ar | MB-276B | -121.710335 | 48.8006803 | 54.7542751 | 1.005 | 0.017 | Andesite | V | Kulshan caldera suite | Hildreth et al., 2003 |
| Wr K-Ar | MB-93 | -121.871016 | 48.86174231 | 36.89636533 | 0.878 | 0.018 | Andesite | V | Early extracaldera lava flows from Mt Baker | Hildreth et al., 2003 |
| Wr K-Ar | MB-91 | -121.944213 | 48.81745383 | 28.76138252 | 0.859 | 0.014 | Andesite | V | Early extracaldera lava flows from Mt Baker | Hildreth et al., 2003 |
| Wr K-Ar | MB-81 | -121.722576 | 48.8547952 | 53.39378926 | 0.743 | 0.034 | Andesite | V | Early extracaldera lava flows from Mt Baker | Hildreth et al., 2003 |
| Wr K-Ar | MB-640 | -121.751692 | 48.7804437 | 50.15788814 | 0.743 | 0.072 | Andesite | V | Early extracaldera lava flows from Mt Baker | Hildreth et al., 2003 |
| Wr K-Ar | MB-52 | -121.851179 | 48.71580346 | 39.10098866 | 0.716 | 0.045 | Basalt | V | Early extracaldera lava flows from Mt Baker | Hildreth et al., 2003 |
| Wr K-Ar | MB-225 | -121.810782 | 48.87193235 | 43.59068974 | 0.613 | 0.008 | Dacite | V | Early extracaldera lava flows from Mt Baker | Hildreth et al., 2003 |
| Wr K-Ar | MB-99 | -121.748943 | 48.83619194 | 50.46346304 | 0.515 | 0.008 | Andesite | V | Oldest unaltered intracaldera andesitic lava flow | Hildreth et al., 2003 |
| Wr K-Ar | MB-135 | -121.756022 | 48.82547284 | 49.67663871 | 0.457 | 0.040 | Andesite | V | Oldest unaltered intracaldera andesitic lava flow | Hildreth et al., 2003 |
| Wr K-Ar | MB-67 | -121.895581 | 48.73692941 | 34.16627492 | 0.495 | 0.018 | Andesite | V | Black Buttes stratovolcano | Hildreth et al., 2003 |
| Wr K-Ar | MB-63 | -121.892822 | 48.73870224 | 34.47292075 | 0.296 | 0.020 | Andesite | V | Black Buttes stratovolcano | Hildreth et al., 2003 |
| Wr K-Ar | MB-116 | -121.884404 | 48.75031652 | 35.40848586 | 0.374 | 0.010 | Andesite | V | Black Buttes stratovolcano | Hildreth et al., 2003 |
| Wr K-Ar | MB-246 | -121.882644 | 48.76829254 | 35.60408894 | 0.346 | 0.009 | Andesite | V | Black Buttes stratovolcano | Hildreth et al., 2003 |
| Wr K-Ar | MB-603 | -121.881443 | 48.76108368 | 35.73750709 | 0.343 | 0.005 | Andesite | V | Black Buttes stratovolcano | Hildreth et al., 2003 |
| Wr K-Ar | MB-249 | -121.908457 | 48.77033914 | 32.73523793 | 0.339 | 0.011 | Andesite | V | Black Buttes stratovolcano | Hildreth et al., 2003 |
| Wr K-Ar | MB-49 | -121.807494 | 48.72345666 | 43.95615923 | 0.336 | 0.015 | Andesite | V | Black Buttes stratovolcano | Hildreth et al., 2003 |
| Wr K-Ar | MB-119 | -121.92406 | 48.74079732 | 31.00112922 | 0.334 | 0.015 | Andesite | V | Black Buttes stratovolcano | Hildreth et al., 2003 |
| Wr K-Ar | MB-238 | -121.872897 | 48.77809383 | 36.68729064 | 0.334 | 0.011 | Andesite | V | Black Buttes stratovolcano | Hildreth et al., 2003 |
| Wr K-Ar | MB-16 | -121.882363 | 48.78088494 | 35.63522097 | 0.306 | 0.013 | Andesite | V | Black Buttes stratovolcano | Hildreth et al., 2003 |
| Wr K-Ar | MB-110 | -121.874701 | 48.75831911 | 36.48678255 | 0.288 | 0.015 | Andesite | V | Black Buttes stratovolcano | Hildreth et al., 2003 |
| Wr K-Ar | MB-615 | -121.733933 | 48.78294894 | 52.13166032 | 0.460 | 0.013 | Andesite | V | Satellite centers contemporaneous with Black Buttes | Hildreth et al., 2003 |
| Wr K-Ar | MB-61 | -121.744933 | 48.77857099 | 50.90913676 | 0.296 | 0.015 | Andesite | V | Satellite centers contemporaneous with Black Buttes | Hildreth et al., 2003 |
| Wr K-Ar | MB-22 | -121.722492 | 48.69735533 | 53.40315038 | 0.455 | 0.009 | Andesite | V | Satellite centers contemporaneous with Black Buttes | Hildreth et al., 2003 |
| Wr K-Ar | MB-222 | -121.773225 | 48.73479255 | 47.76478584 | 0.366 | 0.010 | Andesite | V | Satellite centers contemporaneous with Black Buttes | Hildreth et al., 2003 |
| Wr K-Ar | MB-151 | -121.847679 | 48.8093322 | 39.49001295 | 0.322 | 0.012 | Andesite | V | Satellite centers contemporaneous with Black Buttes | Hildreth et al., 2003 |
| Wr K-Ar | MB-589 | -121.839592 | 48.8056525 | 40.38881016 | 0.322 | 0.009 | Andesite | V | Satellite centers contemporaneous with Black Buttes | Hildreth et al., 2003 |
| Wr K-Ar | MB-223 | -121.786263 | 48.87077718 | 46.31572544 | 0.334 | 0.009 | Andesite | V | Satellite centers contemporaneous with Black Buttes | Hildreth et al., 2003 |
| Wr K-Ar | MB-59 | -121.846814 | 48.72835501 | 39.58617675 | 0.333 | 0.012 | Andesite | V | Satellite centers contemporaneous with Black Buttes | Hildreth et al., 2003 |
| Wr K-Ar | MB-50 | -121.846958 | 48.72205888 | 39.57017129 | 0.331 | 0.018 | Basalt | V | Satellite centers contemporaneous with Black Buttes | Hildreth et al., 2003 |
| Wr K-Ar | MB-89 | -121.741175 | 48.81991379 | 51.32680857 | 0.305 | 0.006 | Andesite | V | Ptarmigan Ridge and Table Mountain lavas | Hildreth et al., 2003 |
| Wr K-Ar | MB-89 | -121.738428 | 48.8207834 | 51.63205273 | 0.306 | 0.013 | Andesite | V | Ptarmigan Ridge and Table Mountain lavas | Hildreth et al., 2003 |
| Wr K-Ar | MB-391 | -121.682982 | 48.85704853 | 57.79423382 | 0.301 | 0.005 | Andesite | V | Ptarmigan Ridge and Table Mountain lavas | Hildreth et al., 2003 |
| Wr K-Ar | MB-10 | -121.698185 | 48.84912447 | 56.10465285 | 0.309 | 0.013 | Andesite | V | Ptarmigan Ridge and Table Mountain lavas | Hildreth et al., 2003 |
| Wr K-Ar | MB-488 | -121.717401 | 48.84394205 | 53.96898404 | 0.189 | 0.011 | Andesite | V | Ptarmigan Ridge and Table Mountain lavas | Hildreth et al., 2003 |
| Wr K-Ar | MB-262 | -121.816703 | 48.85220125 | 42.93263205 | 0.192 | 0.008 | Andesite | V | Younger lavas inset along Cougar Divide | Hildreth et al., 2003 |
| Wr K-Ar | MB-260 | -121.818448 | 48.83602562 | 42.73874985 | 0.119 | 0.023 | Andesite | V | Younger lavas inset along Cougar Divide | Hildreth et al., 2003 |
| Wr K-Ar | MB-335 | -121.810038 | 48.84583517 | 43.67335604 | 0.105 | 0.008 | Andesite | V | Younger lavas inset along Cougar Divide | Hildreth et al., 2003 |
| Wr K-Ar | MB-53 | -121.848482 | 48.71487688 | 39.40082802 | 0.203 | 0.025 | Andesite | V | Younger mafic outliers | Hildreth et al., 2003 |
| Wr K-Ar | MB-175B | -121.729466 | 48.63625504 | 52.62810201 | 0.094 | 0.021 | Basalt | V | Younger mafic outliers | Hildreth et al., 2003 |
| Wr K-Ar | MB-193 | -121.695911 | 48.72674642 | 56.35735248 | 0.199 | 0.005 | Rhyodacite | V | Boulder Ridge lavas | Hildreth et al., 2003 |
| Wr K-Ar | MB-645 | -121.762823 | 48.77066753 | 48.92082738 | 0.140 | 0.055 | Andesite | V | Boulder Ridge lavas | Hildreth et al., 2003 |
| Wr K-Ar | MB-39 | -121.764405 | 48.76168797 | 48.74504171 | 0.090 | 0.052 | Andesite | V | Boulder Ridge lavas | Hildreth et al., 2003 |
| Wr K-Ar | MB-402 | -121.761529 | 48.76795468 | 49.0646607 | 0.080 | 0.014 | Andesite | V | Boulder Ridge lavas | Hildreth et al., 2003 |
| Wr K-Ar | MB-12 | -121.793685 | 48.90234227 | 45.49086856 | 0.202 | 0.009 | Andesite | V | Intracanyon lava flows | Hildreth et al., 2003 |
| Wr K-Ar | MB-332A | -121.792169 | 48.90862381 | 45.65937696 | 0.149 | 0.005 | Dacite | V | Intracanyon lava flows | Hildreth et al., 2003 |
| Wr K-Ar | MB-92 | -121.810141 | 48.89891476 | 43.66194695 | 0.114 | 0.009 | Andesite | V | Intracanyon lava flows | Hildreth et al., 2003 |
| Wr K-Ar | MB-168 | -121.784605 | 48.82667737 | 46.49996979 | 0.076 | 0.007 | Andesite | V | Intracanyon lava flows | Hildreth et al., 2003 |
| Wr K-Ar | MB-232 | -121.755107 | 48.80747006 | 49.77843325 | 0.070 | 0.007 | Andesite | V | Intracanyon lava flows | Hildreth et al., 2003 |
| Wr K-Ar | MB-572 | -121.677945 | 48.78951755 | 58.35403441 | 0.048 | 0.018 | Andesite | V | Intracanyon lava flows | Hildreth et al., 2003 |
| Wr K-Ar | MB-507 | -121.86605 | 48.71954932 | 37.44830161 | 0.043 | 0.005 | Andesite | V | Mount Baker Stratovolcano | Hildreth et al., 2003 |
| Wr K-Ar | MB-520 | -121.815861 | 48.71454637 | 43.02619053 | 0.036 | 0.014 | Andesite | V | Mount Baker Stratovolcano | Hildreth et al., 2003 |
| Wr K-Ar | MB-524 | -121.841644 | 48.71660768 | 40.16070948 | 0.032 | 0.014 | Andesite | V | Mount Baker Stratovolcano | Hildreth et al., 2003 |
| Wr K-Ar | MB-458 | -121.869629 | 48.80235234 | 37.05053313 | 0.024 | 0.016 | Andesite | V | Mount Baker Stratovolcano | Hildreth et al., 2003 |
| Wr K-Ar | MB-632 | -121.888571 | 48.80793439 | 34.94531285 | 0.014 | 0.009 | Andesite | V | Mount Baker Stratovolcano | Hildreth et al., 2003 |
| Wr K-Ar | MB-46 | -121.815227 | 48.74152949 | 43.09665621 | 0.011 | 0.009 | Andesite | V | Mount Baker Stratovolcano | Hildreth et al., 2003 |
| Wr K-Ar | MB-43 | -121.849204 | 48.74277333 | 39.3204983 | 0.009 | 0.011 | Andesite | V | Mount Baker Stratovolcano | Hildreth et al., 2003 |
| Wr K-Ar | MB-294 | -121.853373 | 48.79859321 | 38.85724294 | 0.009 | 0.007 | Andesite of Roosevelt Glacier | V | Mount Baker Stratovolcano | Hildreth et al., 2003 |
| Hb K-Ar | RWT 469A-67 | -121.524993 | 48.86413266 | 75.35299663 | 3.6 | 1.0 | Andesite | V | Hannegan Pass | Engels et al., 1976 |
| Hb K-Ar | RWT 469B-67 | -121.524964 | 48.86512286 | 75.35623356 | 3.3 | 1.0 | Andesite | V | Hannegan Pass | Engels et al., 1976 |
| Pl Ar-Ar | MB-618 | -121.742559 | 48.81902926 | 51.1729383 | 0.992 | 0.014 | Rhyodacite | V | Mount Baker Volcanic Field | Hildreth et al., 2003 |
| Pl Ar-Ar | MB-628 | -121.813914 | 48.8548715 | 43.24266407 | 1.015 | 0.018 | Rhyodacite | V | Mount Baker Volcanic Field | Hildreth et al., 2003 |
| Pl Ar-Ar | MB-212 | -121.705416 | 48.83301237 | 55.30095361 | 1.012 | 0.008 | Rhyodacite | V | Mount Baker Volcanic Field | Hildreth et al., 2003 |
| Pl Ar-Ar | MB-212 | -121.705416 | 48.83301237 | 55.30095361 | 0.994 | 0.015 | Rhyodacite | V | Mount Baker Volcanic Field | Hildreth et al., 2003 |
| Pl Ar-Ar | MB-212 mean | -121.705416 | 48.83301237 | 55.30095361 | 1.008 | 0.007 | Rhyodacite | V | Mount Baker Volcanic Field | Hildreth et al., 2003 |
| Pl Ar-Ar | MB-145 | -121.836368 | 48.82721147 | 40.74709451 | 1.018 | 0.008 | Rhyodacite | V | Mount Baker Volcanic Field | Hildreth et al., 2003 |
| Pl Ar-Ar | MB-82-2 | -121.71858 | 48.85115228 | 53.83797894 | 1.063 | 0.012 | Rhyodacite | V | Mount Baker Volcanic Field | Hildreth et al., 2003 |
| Pl Ar-Ar | MB-630 | -121.727076 | 48.83865146 | 52.8937021 | 1.041 | 0.011 | Rhyodacite | V | Mount Baker Volcanic Field | Hildreth et al., 2003 |
| Pl Ar-Ar | MB-630 | -121.727076 | 48.83865146 | 52.8937021 | 1.073 | 0.016 | Rhyodacite | V | Mount Baker Volcanic Field | Hildreth et al., 2003 |
| Pl Ar-Ar | MB-630 mean | -121.705416 | 48.83301237 | 55.30095361 | 1.008 | 0.007 | Rhyodacite | V | Mount Baker Volcanic Field | Hildreth et al., 2003 |
| Pl Ar-Ar | MB-287 | -121.723401 | 48.82241724 | 53.30218327 | 1.109 | 0.019 | Rhyodacite | V | Mount Baker Volcanic Field | Hildreth et al., 2003 |
| Pl Ar-Ar | MB-287 | -121.723401 | 48.82241724 | 53.30218327 | 1.111 | 0.015 | Rhyodacite | V | Mount Baker Volcanic Field | Hildreth et al., 2003 |
| Pl Ar-Ar | MB-287 mean | -121.727076 | 48.83865146 | 52.8937021 | 1.052 | 0.016 | Rhyodacite | V | Mount Baker Volcanic Field | Hildreth et al., 2003 |
| Pl Ar-Ar | MB-227 | -121.730484 | 48.81169973 | 52.51498173 | 1.114 | 0.011 | Rhyodacite | V | Mount Baker Volcanic Field | Hildreth et al., 2003 |
| Pl Ar-Ar | MB-227 | -121.730484 | 48.81169973 | 52.51498173 | 1.138 | 0.010 | Rhyodacite | V | Mount Baker Volcanic Field | Hildreth et al., 2003 |
| Pl Ar-Ar | MB-227 mean | -121.723401 | 48.82241724 | 53.30218327 | 1.110 | 0.012 | Rhyodacite | V | Mount Baker Volcanic Field | Hildreth et al., 2003 |
| Pl Ar-Ar | MB-188 | -121.696986 | 48.84281344 | 56.23792518 | 1.149 | 0.010 | Rhyodacite | V | Mount Baker Volcanic Field | Hildreth et al., 2003 |
| Pl Ar-Ar | MB-583 | -121.813637 | 48.86656395 | 43.27343013 | 1.151 | 0.016 | Rhyodacite | V | Mount Baker Volcanic Field | Hildreth et al., 2003 |
| Pl Ar-Ar | MB-275C | -121.71435 | 48.80342409 | 54.30803001 | 1.180 | 0.017 | Rhyodacite | V | Mount Baker Volcanic Field | Hildreth et al., 2003 |
| Pl Ar-Ar | MB-626 | -121.827901 | 48.83972068 | 41.68815988 | 1.293 | 0.016 | Rhyodacite | V | Mount Baker Volcanic Field | Hildreth et al., 2003 |
| Pl Ar-Ar | MB-380 | -121.536133 | 48.89036302 | 74.11497719 | 3.722 | 0.020 | Ignimbrite | V | Mount Baker Volcanic Field | Hildreth et al., 2003 |
| Pl Ar-Ar | MB-201 | -121.789859 | 48.83482942 | 45.91611361 | 0.969 | 0.029 | Felsite breccia | V | Mount Baker Volcanic Field | Hildreth et al., 2003 |
| Active volcano | - | -121.81 | 48.77 | 43.677627 | 0.010 | - | - | - | Mount Baker Volcano | - |
| Pl Ar-Ar | MB-636A | -121.79293 | 48.82046733 | 45.57479774 | 0.674 | 0.023 | Granodiorite | I | Mount Baker Volcanic Field | Hildreth et al., 2003 |
| Bt Ar-Ar | MB-333 | -121.642704 | 48.83409025 | 62.27077326 | 2.750 | 0.130 | Lake Ann stock | I | Mount Baker Volcanic Field | Hildreth et al., 2003 |
| Bt K-Ar | RWT 480-67 | -121.631287 | 48.82913807 | 63.53957821 | 2.7 | 0.3 | Lake Ann stock | I | Chilliwack Composite Batholith | Engels et al., 1976 |
| Bt K-Ar | DFC 1-69 | -121.633725 | 48.83610637 | 63.26863455 | 2.5 | 0.1 | Hornfels | I | Chilliwack Composite Batholith | Engels et al., 1976 |
| Wr K-Ar | MB-356 | -121.794313 | 48.81958217 | 45.42102482 | 0.764 | 0.163 | Granodiorite | I | Kulshan caldera suite | Hildreth et al., 2003 |

**Supplementary Table 1c – Summary of apatite fission tracks (AFT) ages around the Osorno and Tronador volcanoes, Southern Andes (data inside the inset of Fig. 1c, plotted in the central panel of Fig. 1d).** W-E distance corresponds to the horizontal distance between each sample and the western boundary of the inset in Fig. 1c. Other columns like in Table 1a.

| **Method** | **Name** | **Longitude** | **Latitude** | **Elevation** | **W-E distance (km)** | **Age (Ma)** | **Error (Ma)** | **Rock** | **Type** | **Place of sampling** | **Reference** |
| --- | --- | --- | --- | --- | --- | --- | --- | --- | --- | --- | --- |
| AFT | AA1 | -72.6812 | -41.2194 | 80 | 19.5 | 24.9 | 4.7 | Granodiorite | I | Lago Llanquihue | Adriasola et al., 2006 |
| AFT | AA102 | -72.4267 | -41.154 | 50 | 47.8 | 6.3 | 2.2 | Tonalite | I | Petrohué | Adriasola et al., 2006 |
| AFT | AA106 | -72.4710 | -42.039 | 5 | 42.9 | 4.3 | 1.5 | Tonalite | I | Isla Pelada | Adriasola et al., 2006 |
| AFT | AA11 | -72.3328 | -41.461 | 150 | 58.3 | 3.3 | 1.2 | Granodiorite | I | Estancia Reloncaví | Adriasola et al., 2006 |
| AFT | AA111 | -72.4903 | -42.022 | 5 | 40.8 | 4.4 | 1.2 | Aplite | I | Isla Pelada | Adriasola et al., 2006 |
| AFT | AA112 | -72.0054 | -41.0885 | 850 | 94.6 | 10.6 | 1.3 | Tonalite | I | Volcan Casablanca | Adriasola et al., 2006 |
| AFT | AA119 | -72.4939 | -42.1683 | 5 | 40.4 | 4.9 | 1.7 | Mica-schist | M | Isla Llancahué | Adriasola et al., 2006 |
| AFT | AA12 | -72.3493 | -41.488 | 180 | 56.4 | 4 | 1.3 | Tonalite | I | Canutillar | Adriasola et al., 2006 |
| AFT | AA125 | -72.3946 | -41.9462 | 100 | 51.4 | 6.5 | 1.2 | Granite | I | Rio Blanco | Adriasola et al., 2006 |
| AFT | AA129 | -72.2897 | -41.11 | 5 | 63.0 | 4.4 | 0.6 | Granite | I | Lago Todos Los Santos | Adriasola et al., 2006 |
| AFT | AA13 | -72.4092 | -41.501 | 300 | 49.8 | 4.8 | 0.8 | Tonalite | I | Lago Chapo | Adriasola et al., 2006 |
| AFT | AA130 | -72.1242 | -41.0738 | 5 | 81.4 | 6.1 | 0.8 | Granite | I | Lago Todos Los Santos | Adriasola et al., 2006 |
| AFT | AA131 | -72.0718 | -41.148 | 20 | 87.3 | 6.9 | 1.3 | Tonalite | I | Lago Todos Los Santos | Adriasola et al., 2006 |
| AFT | AA132 | -72.1755 | -41.0975 | 10 | 75.7 | 7.2 | 1.8 | Tonalite | I | Lago Todos Los Santos | Adriasola et al., 2006 |
| AFT | AA2 | -72.3042 | -41.3782 | 30 | 61.4 | 4.4 | 0.5 | Tonalite | I | Ralún | Adriasola et al., 2006 |
| AFT | AA20 | -72.6417 | -41.4456 | 200 | 23.9 | 11.1 | 1.8 | Granodiorite | I | Correntoso | Adriasola et al., 2006 |
| AFT | AA21 | -72.6331 | -41.4102 | 250 | 24.9 | 11.4 | 1.5 | Granodiorite | I | Lago Chapo | Adriasola et al., 2006 |
| AFT | AA22 | -72.5813 | -41.4383 | 300 | 30.6 | 10.9 | 1.1 | Granodiorite | I | Lago Chapo | Adriasola et al., 2006 |
| AFT | AA23 | -72.6090 | -41.514 | 450 | 27.6 | 8 | 1 | Gneiss | M | Lago Chaiquenes | Adriasola et al., 2006 |
| AFT | AA24 | -72.6998 | -41.5955 | 10 | 17.5 | 9.4 | 2 | Granodiorite | I | Punta Metri | Adriasola et al., 2006 |
| AFT | AA25 | -72.7230 | -41.566 | 20 | 14.9 | 12.5 | 1.6 | Tonalite | I | Punta Metri | Adriasola et al., 2006 |
| AFT | AA26 | -72.668 | -41.637 | 10 | 21.0 | 7.8 | 0.9 | Tonalite | I | Caleta Larenas | Adriasola et al., 2006 |
| AFT | AA27 | -72.626 | -41.692 | 200 | 25.7 | 9.3 | 0.8 | Tonalite | I | Caleta Larenas | Adriasola et al., 2006 |
| AFT | AA29 | -72.2369 | -41.4594 | 150 | 68.9 | 5.5 | 0.9 | Granite | I | Rio Cochamo | Adriasola et al., 2006 |
| AFT | AA3 | -72.349 | -41.5049 | 60 | 56.5 | 5.6 | 2 | Tonalite | I | Estancia Reloncaví | Adriasola et al., 2006 |
| AFT | AA30 | -72.311 | -41.6492 | 100 | 60.7 | 4 | 0.9 | Tonalite | I | Balseo | Adriasola et al., 2006 |
| AFT | AA31 | -72.3008 | -41.6667 | 250 | 61.8 | 4 | 0.9 | Granite | I | Puelo Chico | Adriasola et al., 2006 |
| AFT | AA33 | -72.4672 | -41.728 | 20 | 43.3 | 5.8 | 0.5 | Granodiorite | I | Volcán Yates | Adriasola et al., 2006 |
| AFT | AA34 | -72.4010 | -41.705 | 30 | 50.7 | 4.2 | 0.5 | Tonalite | I | Volcán Yates | Adriasola et al., 2006 |
| AFT | AA35 | -72.363 | -41.354 | 150 | 54.9 | 6.2 | 0.8 | Tonalite | I | Termas de Ralún | Adriasola et al., 2006 |
| AFT | AA36 | -72.5890 | -41.589 | 200 | 2.8 | 8.2 | 1.3 | Gneiss | M | Parque Alerce Andino | Adriasola et al., 2006 |
| AFT | AA37 | -72.5726 | -41.747 | 30 | 31.6 | 6.8 | 1 | Tonalite | I | Chaparrano | Adriasola et al., 2006 |
| AFT | AA39 | -72.4346 | -42.02 | 150 | 46.9 | 5.7 | 1.9 | Tonalite | I | Rio Mariquita | Adriasola et al., 2006 |
| AFT | AA4 | -72.4022 | -41.647 | 20 | 50.6 | 3.8 | 0.9 | Tonalite | I | Estancia Reloncaví | Adriasola et al., 2006 |
| AFT | AA40 | -72.4484 | -42.076 | 40 | 45.4 | 4.3 | 0.7 | Granodiorite | I | Pichanco | Adriasola et al., 2006 |
| AFT | AA42 | -72.4027 | -41.907 | 200 | 50.5 | 4.2 | 2.7 | Tonalite | I | Rio Negro | Adriasola et al., 2006 |
| AFT | AA43 | -72.601 | -41.9908 | 100 | 28.5 | 15.5 | 2.7 | Dacite | I | Pichicolo | Adriasola et al., 2006 |
| AFT | AA44 | -72.3689 | -42.18 | 10 | 54.2 | 3.3 | 0.5 | Tonalite | I | F. Quintupeu | Adriasola et al., 2006 |
| AFT | AA45 | -72.4821 | -42.091 | 5 | 41.7 | 4.3 | 1.2 | Mica-schist | M | Isla Llancahué | Adriasola et al., 2006 |
| AFT | AA6 | -72.3424 | -41.5378 | 20 | 57.2 | 3.8 | 0.9 | Tonalite | I | Estancia Reloncaví | Adriasola et al., 2006 |
| AFT | AA8 | -72.3201 | -41.6024 | 80 | 59.7 | 3.3 | 0.6 | Granodiorite | I | Estancia Reloncaví | Adriasola et al., 2006 |
| AFT | AA9 | -72.3003 | -41.5672 | 110 | 61.9 | 3.5 | 0.7 | Granite | I | Estancia Reloncaví | Adriasola et al., 2006 |
| AFT | AL122 | -72.0588 | -41.3606 | 1600 | 88.7 | 6.7 | 1.6 | Tonalite | I | Rio Cochamo | Adriasola et al., 2006 |
| AFT | AL137 | -72.46 | -41.5885 | 1200 | 44.1 | 9.8 | 1.9 | Granodiorite | I | Puelo | Adriasola et al., 2006 |
| AFT | AL152 | -72.0588 | -41.3606 | 1550 | 88.7 | 6.7 | 1.7 | Tonalite | I | Llanada Grande | Adriasola et al., 2006 |
| AFT | SP06b | -72.415 | -41.4933 | 400 | 49.1 | 5.9 | 0.6 | - | - | - | Thomson et al., 2010 |
| AFT | SP11 | -72.6 | -41.73 | 25 | 28.6 | 5.1 | 1 | - | - | - | Thomson et al., 2010 |
| AFT | SP040 | -71.898 | -41.8 | 1675 | 106.5 | 13 | 2.2 | - | - | - | Thomson et al., 2010 |
| AFT | SP041 | -71.888 | -41.862 | 1525 | 107.7 | 14.2 | 3.3 | - | - | - | Thomson et al., 2010 |
| AFT | SP046 | -72.13 | -42.062 | 1450 | 80.8 | 3.4 | 0.5 | - | - | - | Thomson et al., 2010 |
| AFT | SP048 | -71.928 | -41.903 | 275 | 103.2 | 14.1 | 2.7 | - | - | - | Thomson et al., 2010 |
| AFT | SP057 | -72.03 | -41.395 | 1325 | 91.9 | 3.5 | 1.1 | - | - | - | Thomson et al., 2010 |
| AFT | SP061 | -72.337 | -41.995 | 1650 | 57.8 | 3.9 | 0.7 | - | - | - | Thomson et al., 2010 |
| AFT | SP062 | -72.33 | -42.072 | 1325 | 58.6 | 5.1 | 0.6 | - | - | - | Thomson et al., 2010 |
| AFT | SP066 | -72.373 | -41.947 | 125 | 53.8 | 2 | 0.8 | - | - | - | Thomson et al., 2010 |
| AFT | SP067 | -72.647 | -41.408 | 250 | 23.4 | 10.2 | 1.5 | - | - | - | Thomson et al., 2010 |
| AFT | SP128 | -71.568 | -42.03 | 300 | 143.2 | 21 | 2 | - | - | - | Thomson et al., 2010 |
| AFT | SP133 | -71.43 | -41.098 | 250 | 158.6 | 14 | 1.7 | - | - | - | Thomson et al., 2010 |
| AFT | SP137 | -71.777 | -41.578 | 450 | 120.1 | 12.5 | 3.9 | - | - | - | Thomson et al., 2010 |
| AFT | SP139 | -71.612 | -41.593 | 300 | 138.4 | 28.1 | 5.2 | - | - | - | Thomson et al., 2010 |

**Supplementary Table 1d – Summary of isotopic ages < 4 Ma of rocks around the Osorno and Tronador volcanoes, Southern Andes (data inside the inset of Fig. 1c, plotted in the bottom panel of Fig. 1d).** In the field “Method”, **ZFT:** zircon fission tracks, **Wr K-Ar:** ^40^K/^40^Ar in whole rock, **Wr Ar-Ar:** ^40^Ar/^39^Ar in whole rock, **Active volcano:** 0.1 Ma is an arbitrary recent age and the position is according to Venzke (2013). Other columns like in Table 1a.

| **Method** | **Name** | **Longitude** | **Latitude** | **Elevation** | **W-E Horizontal distance (km)** | **Age (Ma)** | **Error (Ma)** | **Rock** | **Type** | **Place of sampling** | **Reference** |
| --- | --- | --- | --- | --- | --- | --- | --- | --- | --- | --- | --- |
| ZFT | AA106 | -72.4710 | -42.0391 | 5 | 42.895 | 3.4 | 0.3 | Tonalite | I | Isla Pelada | Adriasola et al., 2006 |
| ZFT | AA111 | -72.4903 | -42.02187 | 5 | 40.754 | 3.9 | 0.3 | Aplite | I | Isla Pelada | Adriasola et al., 2006 |
| ZFT | AA129 | -72.2897 | -41.11029 | 5 | 63.049 | 3.8 | 0.2 | Granite | I | Lago Todos los Santos | Adriasola et al., 2006 |
| ZFT | AA40 | -72.4484 | -42.07639 | 40 | 45.412 | 3.8 | 0.3 | Granodiorite | I | Pichanco | Adriasola et al., 2006 |
| ZFT | AA45 | -72.4821 | -42.09051 | 5 | 41.670 | 3.2 | 0.5 | Mica-schist | M | Isla Llancahué | Adriasola et al., 2006 |
| ZFT | BR409 | -72.2795 | -41.41409 | 200 | 64.183 | 3.4 | 0.8 | Granite | I | Puente Las Trancas, Ralún | Adriasola et al., 2006 |
| Wr K-Ar | XC-288 | -72.3970 | -40.91695 |  | 51.122 | 0.4 | 0.1 | Basalt | V | Estratos de Chapuco | Lara et al., 2001 |
| Wr K-Ar | XC-289 | -72.3954 | -40.95834 |  | 51.302 | 0.6 | 0.5 | Basalt | V | Estratos de Chapuco | Lara et al., 2001 |
| Wr K-Ar | HO-46 | -72.4952 | -40.95695 |  | 40.205 | 1 | 0.3 | Basalt | V | Estratos de Chapuco | SNGM-BRGM,1995 |
| Wr K-Ar | HO-46 | -72.4952 | -40.95695 |  | 40.205 | 0.9 | 0.3 | Basalt | V | Estratos de Chapuco | Lara et al., 2001 |
| Wr K-Ar | 60385 | -72.5391 | -41.30917 |  | 35.326 | 1.4 | 0.2 | Basalt | V | Estratos de Hueñu Hueñu | Moreno et al.,1985 |
| Wr K-Ar | 090185-3 | -72.2970 | -41.44603 |  | 62.241 | 0.27 | 0.14 | Basalt | V | Estratos de Reloncavi | Moreno et al., 1985 |
| Wr K-Ar | P273b | -72.0661 | -41.3359 |  | 87.897 | 0.7 | 0.4 | basalt | V | Volcán Cuernos del Diablo | SNGM-BRGM, 1995 |
| Wr Ar-Ar | XB-29 | -72.0033 | -41.22027 |  | 94.880 | 0.53 | 0.127 | basalt | V | Tronador Volcanic Group | Mella et al., 2005 |
| Wr Ar-Ar | XB-32 | -71.9675 | -41.18701 |  | 98.860 | 0.47 | 0.036 | basalt | V | Tronador Volcanic Group | Mella et al., 2005 |
| Wr Ar-Ar | XM-7 | -71.8432 | -41.14895 |  | 112.674 | 0.36 | 0.05 | basalt | V | Tronador Volcanic Group | Mella et al., 2005 |
| Wr Ar-Ar | XM-22 | -71.8877 | -41.20374 |  | 107.730 | 0.34 | 0.041 | basalt | V | Tronador Volcanic Group | Mella et al., 2005 |
| Wr K-Ar | XM-23 | -71.8327 | -41.18705 |  | 113.838 | 1.3 | 0.3 | dacite | V | Tronador Volcanic Group | Mella et al., 2005 |
| Active volcano | - | -72,2667 | -40.98333 | 2493 | 65.609 | 0.1 | - | - | - | Puntiagudo Cordon Cenizos | - |
| Active volcano | - | -72,4333 | -41.05 | 1715 | 47.086 | 0.1 | - | - | - | Osorno | - |
| Active volcano | - | -72,5 | -41.1 | 2652 | 39.677 | 0.1 | - | - | - | Osorno | - |
| Active volcano | - | -72,5333 | -41.13333 | 1082 | 35.972 | 0.1 | - | - | - | Osorno | - |
| Active volcano | - | -72,2667 | -41.18333 | 260 | 65.609 | 0.1 | - | - | - | Cayutue La Vigueria | - |
| Active volcano | - | -72,2833 | -41.28333 | 500 | 63.757 | 0.1 | - | - | - | Cayutue La Vigueria | - |
| Active volcano | - | -72,6167 | -41.33333 | 2015 | 26.710 | 0.1 | - | - | - | Cabulco | - |
| Active volcano | - | -72,3833 | -41.78333 | 2111 | 52.643 | 0.1 | - | - | - | Yate | - |
| Active volcano | - | -72,45 | -41.86667 | 1572 | 45.234 | 0.1 | - | - | - | Hornopirén | - |
| Active volcano | - | -72,6167 | -41.88333 | 1670 | 26.710 | 0.1 | - | - | - | Apagado | - |

**Supplementary Table 2 – Material properties used in the numerical experiments.**

|  | ***Ρ_0_^s^, ρ_0_^l^*** | ***E_a_*** | ***V_a_*** | ***n*** | ***C*** | ***Visc.*** | ***Sin*** | ***c*** | ***µ*** | ***C_p_*** | ***Hr*** | ***H_l_*** | ***α*** | ***β*** | ***T_solidus_*** | ***T_liquidus_*** |
| --- | --- | --- | --- | --- | --- | --- | --- | --- | --- | --- | --- | --- | --- | --- | --- | --- |
|  | **(km/m^3^)** | **(kJ/mol)** | **(m^3^/mol)** |  | **(Mpa)** | **flow law** | ***(ϕ_eff_)*** | **(W/m/K)** | **(Gpa)** | **(J/kg/K)** | **(µW/m^3^)** | **(kJ/kg)** | **(1/k)** | **(1/Pa)** | **(K)** | **(K)** |
| **Crust** | 2800 (solidus) 2400 (liquidus) | 154 | 0 | 2.3 | 10 | Wet Qz. | 0.2 | 0.64+807/(T+77) | 10 | 1000 | 1 | 300 | 3x10^-5^ | 1x10^-11^ | 889 + 17900 /(P+54)+ 20200/(P+54)^2^  at P <1200 Mpa;  831 + 0.06 P  at P > 1200 Mpa | 1262 + 0.09 P |
| **Lithospheric mantle** | 3250 (solidus)  2200 (liquidus) | 532 | 10 | 3.5 | 10 | Dry  Ol. | 0.6 | 0.73+1293/(T+77) | 67 | 1000 | 0.022 | 400 | 3x10^-5^ | 1x10^-11^ | 1394 + 0.132899 P-0.000005104P^2^  at P < 1000 Mpa,  2212 + 0.030819 (P-10000)  at P >1000 Mpa | 2073 + 0.114 P |
| **Asthenospheric mantle** | 3250 (solidus) 2200 (liquidus) | 532 | 10 | 3.5 | 10 | Dry  Ol. | 0.6 | 0.73+1293/(T+77) | 67 | 1000 | 0.022 | 400 | 3x10^-5^ | 1x10^-11^ | 1394 + 0.132899 P-0.000005104 P^2^  at P < 1000 Mpa,  2212 + 0.030819 (P-1000)  at P>10000 Mpa | 2073 + 0.114 P |
| **MMZ and 75% of the magma**  **channel** | 3250 (solidus)  1800 (liquidus) | 471 | 0 | 4.0 | 10 | Wet Ol. | 0 | 0.73+1293/(T+77) | 67 | 1000 | 0.022 | 400 | 3x10^-5^ | 1x10^-11^ | 1240 + 49800/(P+323)  at P<2400 Mpa,  1266 + 0.114 P + 0.000005 P^2^  at P>2400 Mpa | 2073 + 0.114 P |
| **Basalt 25% of the magma channel** | 3100 (solidus), 1800 (liquidus) | 154 | 0 | 2.3 | 10 | Wet Qz. | 0 | 1.18+474/(T+77) | 25 | 1000 | 0.25 | 380 | 3x10^-5^ | 1x10^-11^ | 973-70400/(P+354)+77800000  at P<1600  935+0.0035 P + 0.0000062 P^2^  at P>1600 | 1423 + 0.105 P |

$\boldsymbol{\rho}_{\boldsymbol{l}}^{\boldsymbol{0}}$ and $\boldsymbol{\rho}_{\boldsymbol{s}}^{\boldsymbol{0}}$ are the standard densities of solid and molten rocks, respectively; $\boldsymbol{E}_{\boldsymbol{a}}$ is the activation energy; $\boldsymbol{V}_{\boldsymbol{a}}$ is the activation volume; ***n*** is the stress exponent; ***C*** is cohesion; $\boldsymbol{\varphi}$ is the effective internal friction angle; ***C_p_*** is the specific heat capacity; ***c*** is thermal conductivity; $\boldsymbol{\mu}$ is the shear modulus; ***H_r_*** and ***H_l_*** are the radiogenic and latent heat productions, respectively; $\boldsymbol{\alpha}$ is thermal expansion; $\boldsymbol{\beta}$ is compressibility; $\boldsymbol{T}_{\boldsymbol{s}}$ and $\boldsymbol{T}_{\boldsymbol{l}}$ are the solidus and liquidus temperatures, respectively. Qz and Ol are quartzite and olivine, respectively. All rheological and partial melting laws/parameters are based on experimental rock mechanics and petrology (Ranalli, 1995; Hirschmann, 2000; Johannes, 1985; Turcotte, 2002).

**References**

Adriasola, A. C., Thomson, S. N., Brix, M. R., Hervé, F. & Stöckhert, B. Postmagmatic cooling and late Cenozoic denudation of the North Patagonian Batholith in the Los Lagos region of Chile, 41°-42°15′S. *Int. J. Earth Sci.* 95, 504–528 (2006).

Engels, J.C., Tabor, R. W., Miller, F.K., Obradovich, J. D. Summary of K-Ar, Rb-Sr, U-Pb, Pba, and fission-track ages of rocks from Washington State prior to 1975 (exclusive of Columbia Plateau basalts). *Miscellaneous Field Studies Map 710* (1976).

Hildreth, W., Fierstein, J. & Lanphere, M. Eruptive history and geochronology of the Mount Baker volcanic field, Washington. *Bull. Geol. Soc. Am.* 115, 729–764 (2003).

Hirschmann, M. M. Mantle solidus: Experimental constraints and the effects of peridotite composition. *Geochemistry, Geophys. Geosystems* 1, (2000).

Johannes, W. *The significance of experimental studies for the formation of migmatites. in Migmatites* (ed. Ashworth, J. R.) (Blackie & Son Ltd, USA Chapman & Hall, 1985).

Lara, L. ., Rodríguez, C. ., Moreno, H. . & Pérez de Arce, C. Geocronología K-Ar y geoquímica del volcanismo plioceno superior-pleistoceno de los Andes del sur (39-42°S). *Rev. Geol. Chile* 28, (2001).

Mella, M. et al. Petrogenesis of the Pleistocene Tronador Volcanic Group, Andean Southern Volcanic Zone. *Rev. Geol. Chile* 32, 131–154 (2005).

Moreno, H., Varela, J., López, L., Munizaga, F., and Lahsen, A. *Geología y riesgo volcánico del volcán Osorno y centros eruptivos menores: Unpublished report* *No. OICB-06C to ENDESA-CORFO*, 212 p. (1985).

Ranalli, G. *Rheology of Earth.* (Springer Sciences and Business Media, 1995).

Reiners, P. W. et al. Late Miocene exhumation and uplift of the Washington Cascade Range. *Geology* 30, 767–770 (2002).

SERNAGEOMIN-BGRM. *Carta metalogénica X región sur: Servicio Nacional de Geología y Minería.* (1995).

Simon-Labric, T. et al. Low-temperature thermochronologic signature of rangedivide migration and breaching in the North Cascades. *Lithosphere* 6, 473–482 (2014).

Thomson, S. N. et al. Glaciation as a destructive and constructive control on mountain building. *Nature* 467, 313–317 (2010).

Turcotte, D. L. & Schubert, G. *Geodynamics.* (Cambridge University Press, 2002).

Venzke, E. *Global Volcanism Program.* Volcanoes of the World (2013). doi:https://doi.org/10.5479/si.GVP.VOTW4-2013.

**Supplementary** **Figures**


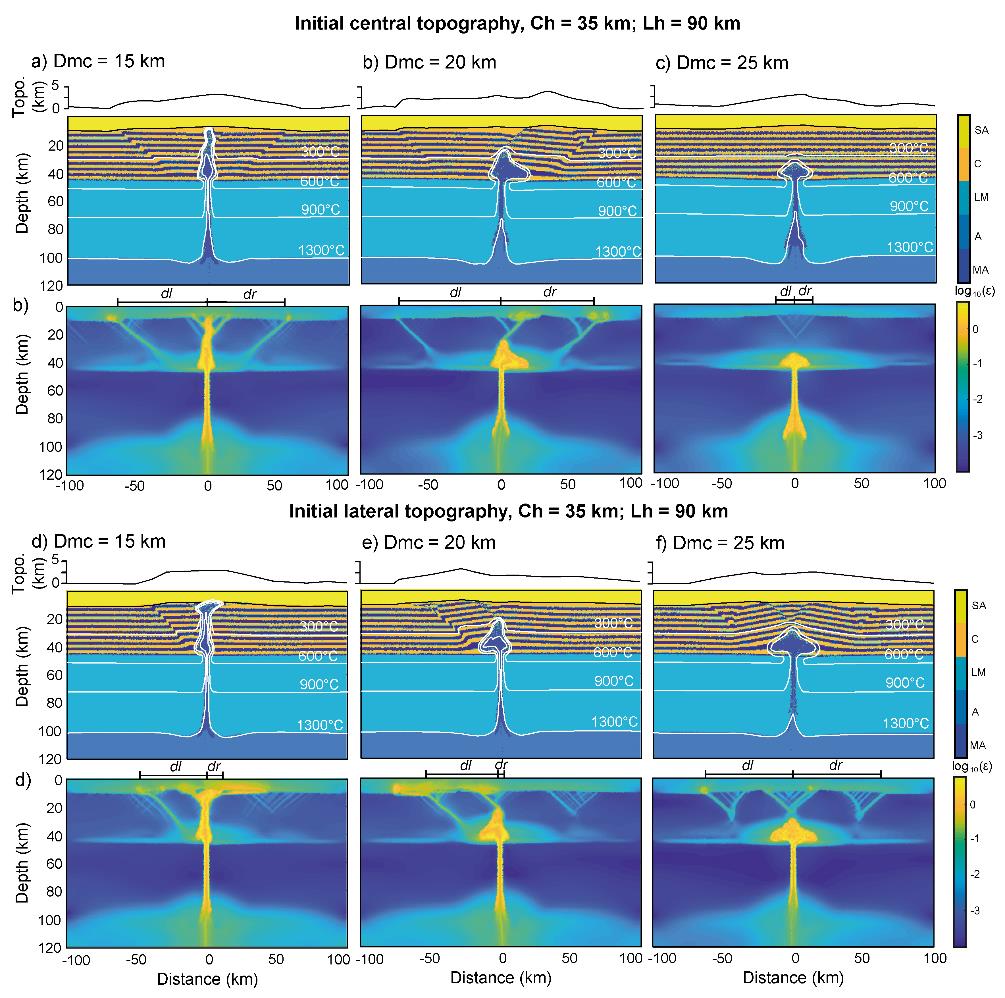


**Supplementary Fig. 1. Final steps of simulations accounting for Ch = 35 km and Lh = 90 km.** Symbols and definitions are the same of Fig. 2. **a-c)** The initial central topography is centered above the MMZ. **d-f)** The initial topography is laterally shifted (rightward) respect to the MMZ.


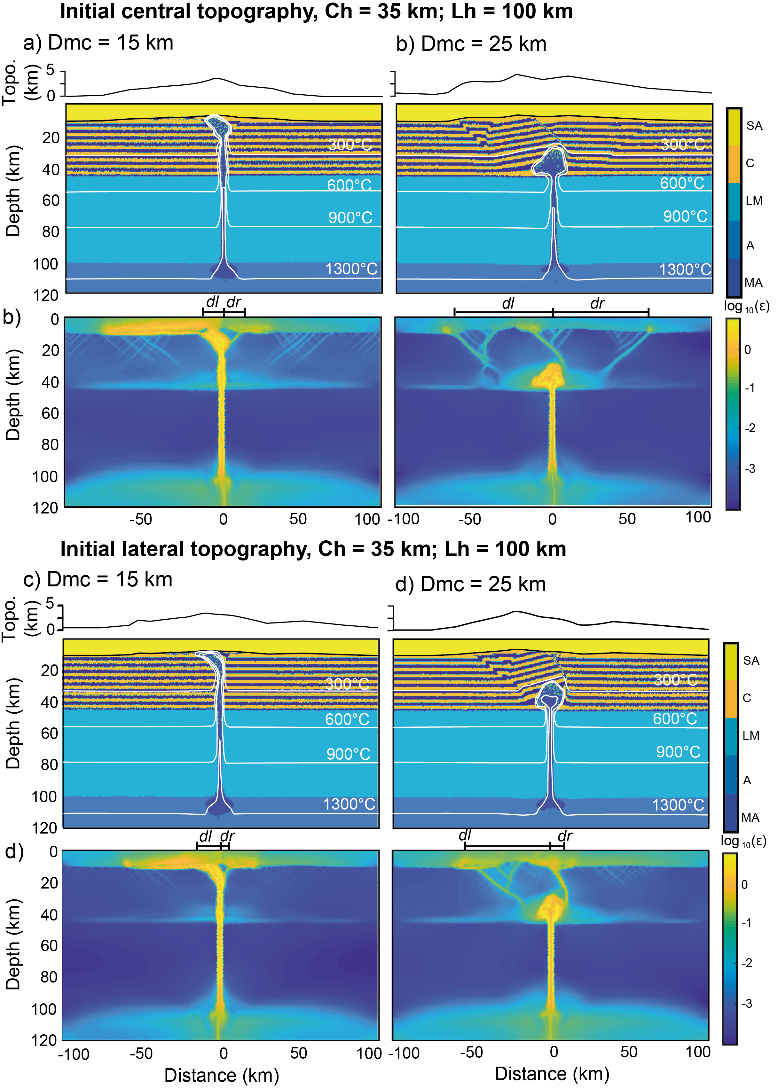


**Supplementary Fig. 2. Final steps of simulations accounting for Ch = 35 km and Lh = 100 km.** Symbols and definitions are the same of Fig. 2. **a, b)** The initial topography is centered above the MMZ. **c, d)** The initial topography is laterally shifted (rightward) respect to the MMZ.


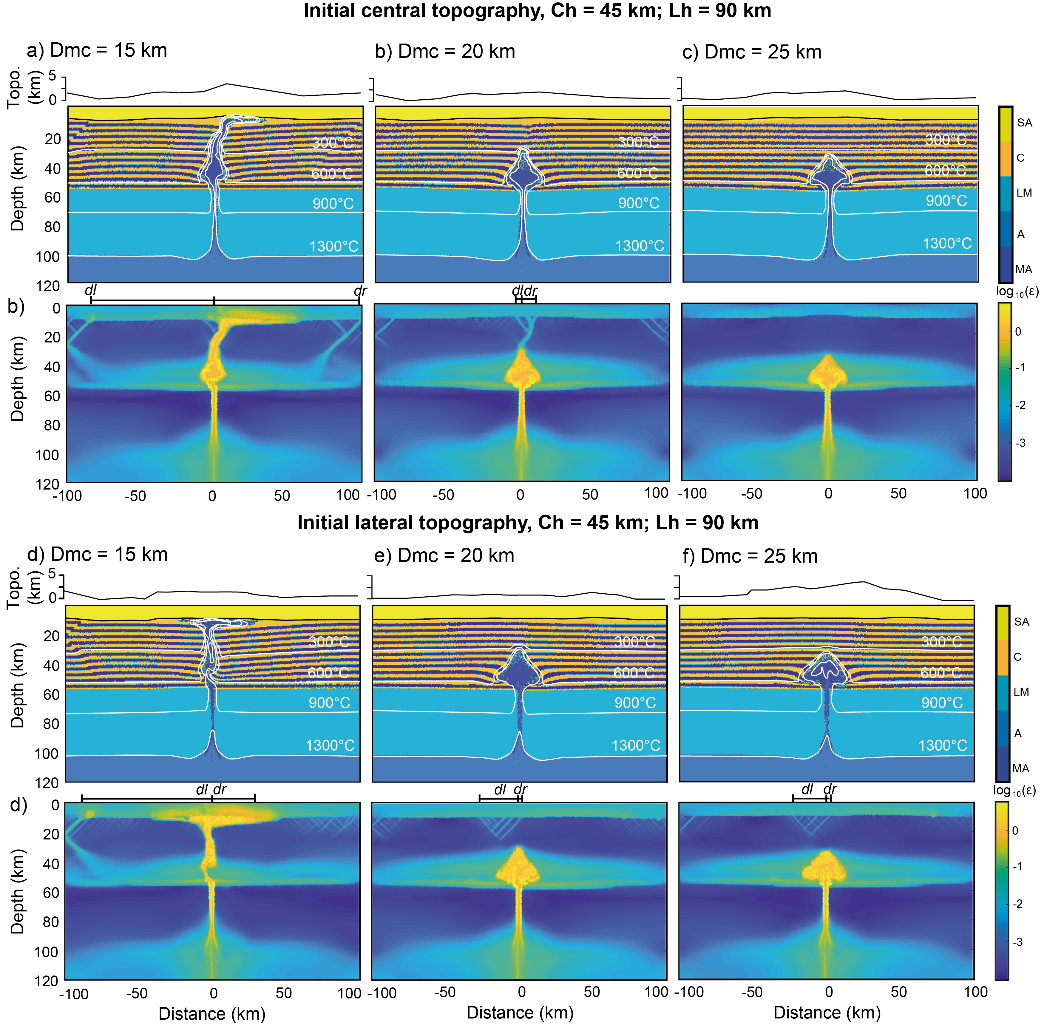


**Supplementary Fig. 3. Final steps of simulations accounting for Ch = 45 km and Lh = 90 km.** Symbols and definitions are the same of Fig. 2. **a-c)** The initial topography is centered above the MMZ. **d-f)** The initial topography is laterally shifted (rightward) respect to the MMZ.


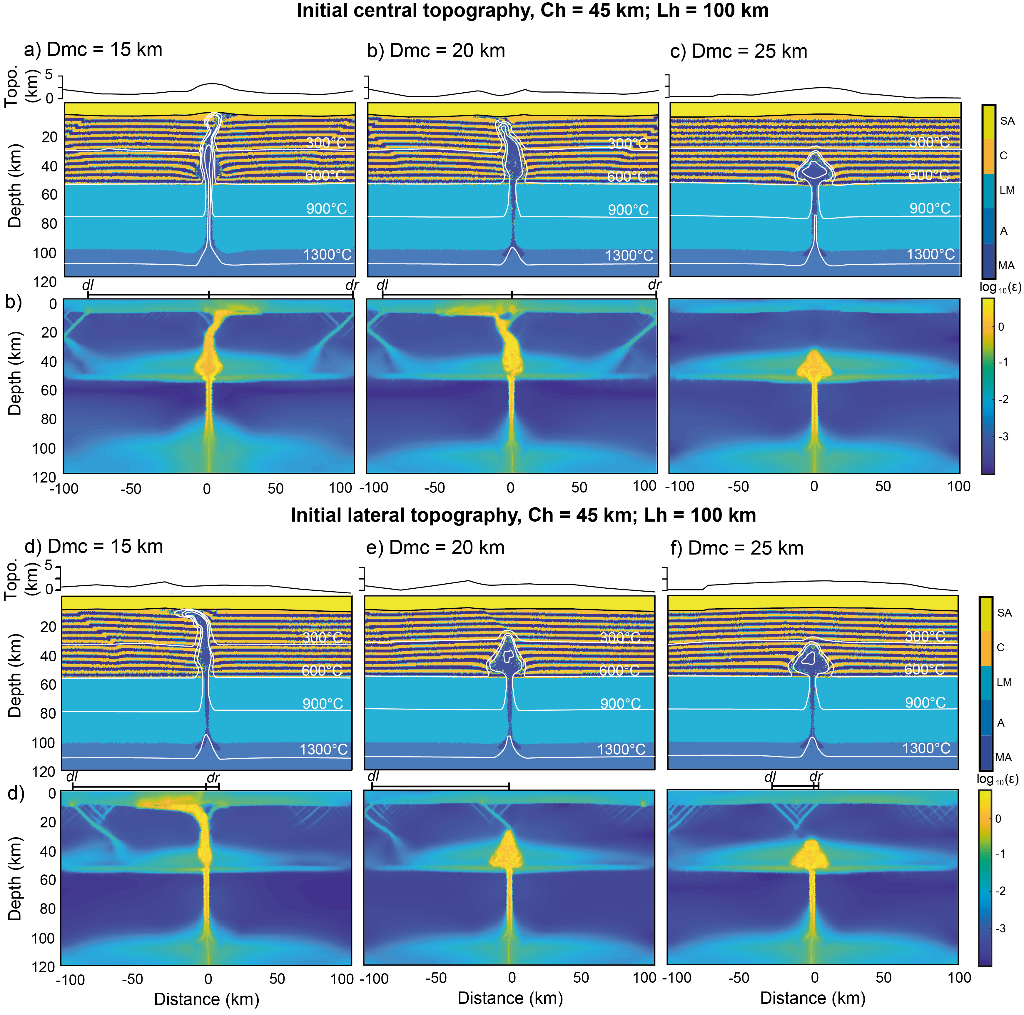


**Supplementary Fig. 4. Final steps of simulations accounting for Ch = 45 km and Lh = 100 km.** Symbols and definitions are the same of Fig. 2. **a-c)** The initial topography is centered above the MMZ. **d-f)** The initial topography is laterally shifted (rightward) respect to the MMZ.


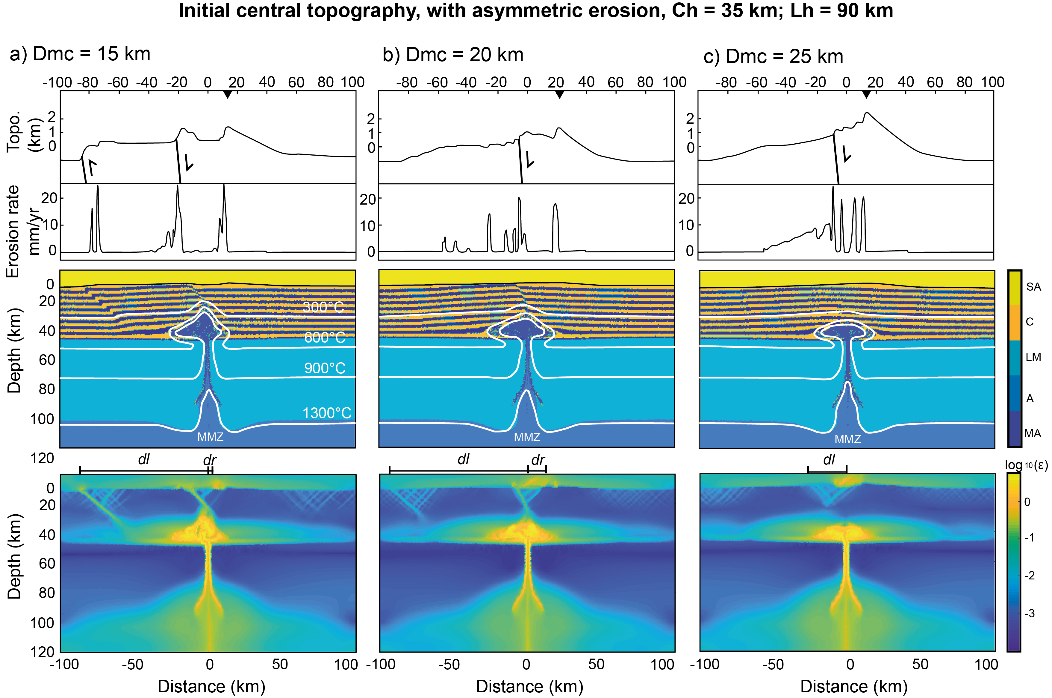


**Supplementary Fig. 5. Final steps of simulations accounting for Ch = 35 km and Lh = 90 km with asymmetric erosion.** Symbols and definitions are the same of Fig. 3. **a-c)** The initial topography is centered above the MMZ.


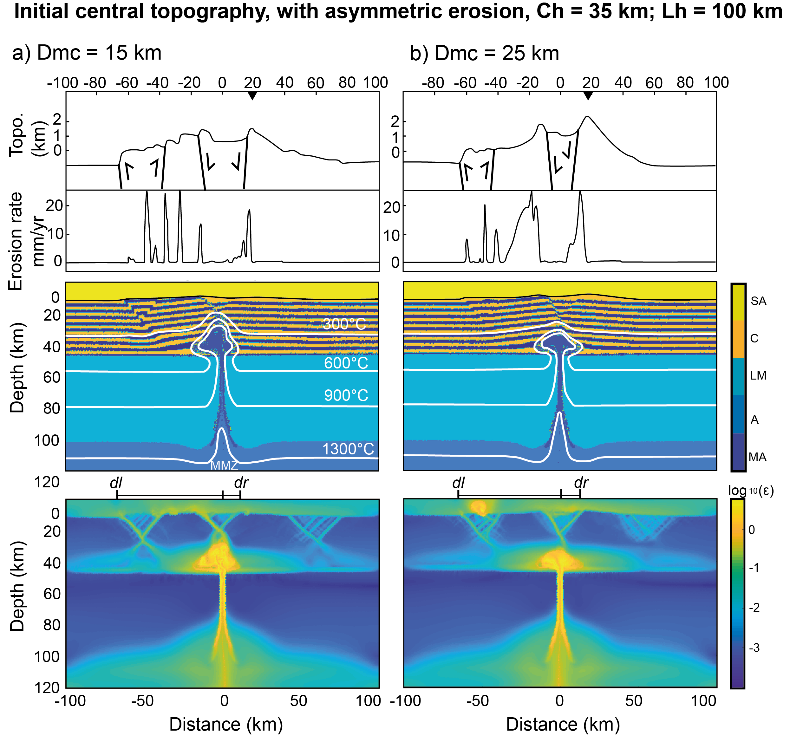


**Supplementary Fig. 6. Final steps of simulations accounting for Ch = 35 km and Lh = 100 km with asymmetric erosion.** Symbols and definitions are the same of Fig. 3. **a-b)** The initial topography is centered above the MMZ.


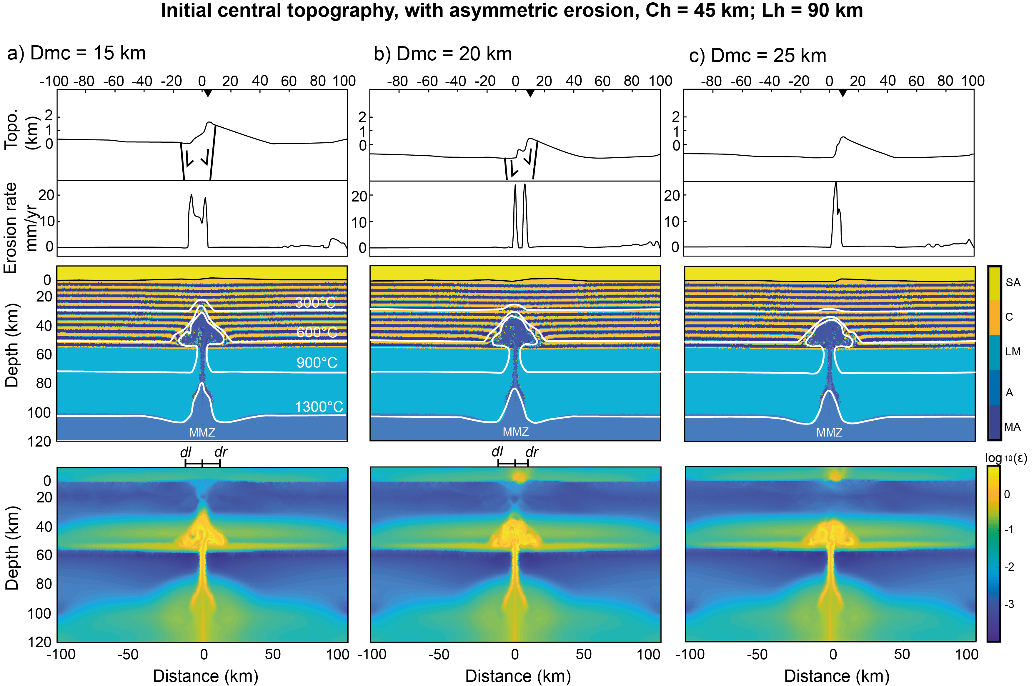


**Supplementary Fig. 7. Final steps of simulations accounting for Ch = 45 km and Lh = 90 km with asymmetric erosion.** Symbols and definitions are the same of Fig. 3. **a-c)** The initial topography is centered above the MMZ.


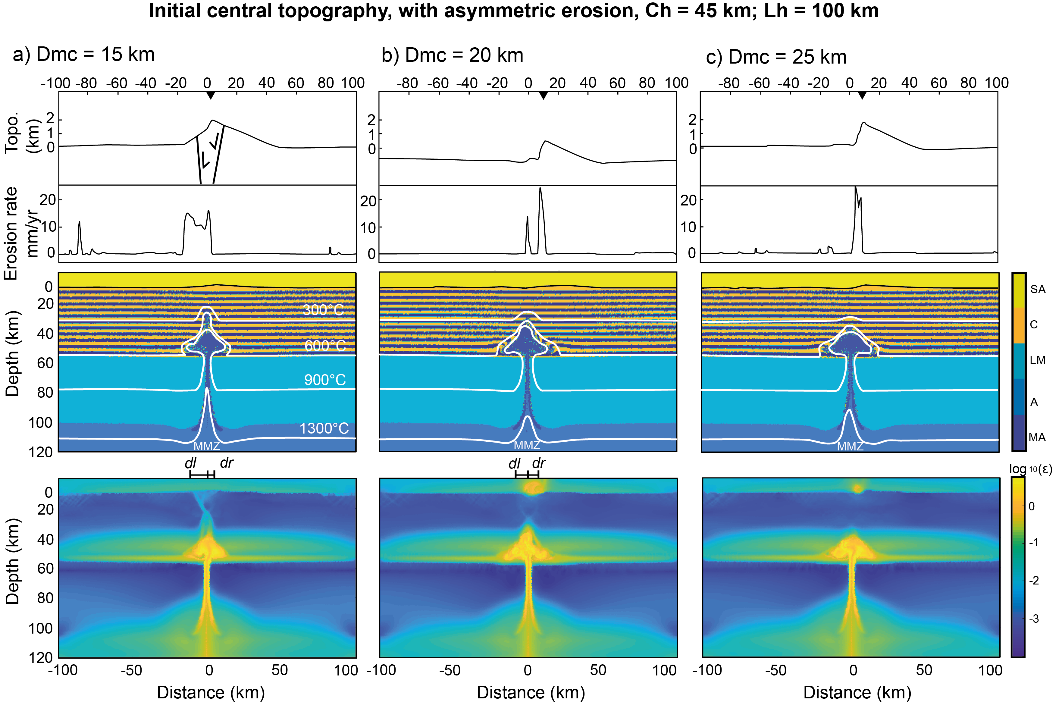


**Supplementary Fig. 8. Final steps of simulations accounting for Ch = 45 km and Lh = 100 km with asymmetric erosion.** Symbols and definitions are the same of Fig. 3. **a-c)** The initial topography is centered above the MMZ.
